# Location-selective immobilisation of single-atom catalysts on the surface or within the interior of ionic nanocrystals using coordination chemistry

**DOI:** 10.1038/s41467-023-40003-8

**Published:** 2023-07-15

**Authors:** Kenichi Endo, Masaki Saruyama, Toshiharu Teranishi

**Affiliations:** 1 Institute for Chemical Research, Kyoto University, Gokasho, Uji, Kyoto, 611-0011 Japan; 2 Present Address: Max Planck Institute for Solid State Research, Heisenbergstr. 1, 70569 Stuttgart, Germany

**Keywords:** Photocatalysis, Coordination chemistry, Quantum dots, Synthesis and processing

## Abstract

Single-atom catalysts dispersed on support materials show excellent heterogeneous catalytic properties that can be tuned using the interactions between the single atoms and the support. Such interactions depend on whether the single atoms are located on the surface or within the interior of the support. However, little is known about immobilising single atoms on the surface or within the interior of supports deliberately and selectively. Herein, such location-selective placement of single atoms is achieved through the choice of metal complex precursor, solvent, and workup procedure. Using CdSe nanoplatelets as a support, a *cis*-[PtCl_2_(SO(CH_3_)_2_)_2_] precursor in an aprotic solvent exclusively attaches single Pt atoms on the surface of the support. In contrast, a [PtCl_4_]^2−^ precursor in a protic solvent followed by amine treatment places 60% of the single Pt atoms inside the support by cation substitution. The surface-adsorbed single Pt atoms show higher stability in photocatalytic hydrogen evolution than the substituted ones, and the preclusion of substitution as internal Pt maximises the activity. Thus, this study provides a viable strategy for the structurally precise synthesis and design of single-atom catalysts.

## Abstract (web summary)

The authors report the location-selective immobilisation of single Pt atoms on the surface or within the interior of CdSe nanoplatelets using coordination chemistry and show location-dependent photocatalytic reactivity.

## Introduction

**Fig. 1:**
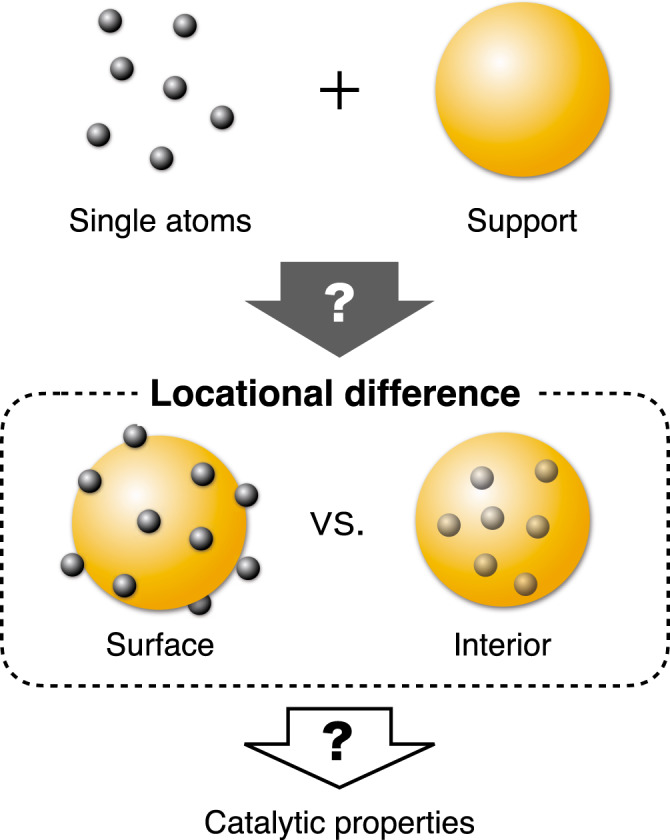
Schematic of the locational difference in SA catalysts. Immobilization of single atoms to a support can occur at surface and/or interior locations, and the different locations leads to different catalytic properties.

Single atoms (SAs) directly immobilised on support materials are promising catalysts because they enable all the active atoms to be utilised on the surface, and their properties can be modulated through their interactions with the support^1–10^. Accordingly, SA catalysts show potential for key catalytic reactions such as the water-gas shift reaction^11^, preferential oxidation of CO in H_2_^12^, and H_2_ evolution^13–16^. In the design of SA catalysts, it is essential to control the environment of each SA in terms of coordination species, coordination number, spatial microenvironment, and spatial location, because these factors affect their interactions with the support material and thus their catalytic properties^17–20^. Specifically, supported SA catalysts can have two distinct spatial locations^21,22^: they can either be attached to the surface of the support by adsorption^14,16,23–26^ or substituted within the interior of the support^15,27–31^ (Fig. 1). These different locations impact the catalytic properties of the SAs owing to differences in both metal–support interactions and surface availability. Nevertheless, little is known about how to control the locations of SAs and how this affects catalytic properties. Therefore, establishing a methodology to influence the adsorption vs. substitution of SAs on/in supports is crucial for the precise design and synthesis of SA catalysts.

Herein, we develop a method for surface/interior-selective loading of SAs and investigate the influence of this locational control on photocatalytic performance. Surface-selective adsorption of Pt SAs on CdSe nanoplatelets (NPLs) is achieved using an aprotic solvent system and *cis*-[PtCl_2_(dmso)_2_] (dmso: dimethyl sulfoxide) as a precursor. The adsorption process is based on the exchange of one dmso ligand with a Se atom at the CdSe surface. Conversely, the preferential substitution of Cd^2+^ with Pt^2+^ in the interior of the NPLs is achieved using a protic solvent system and [PtCl_4_]^2−^ as a precursor. The Pt SA locations can be determined thanks to the atomically defined thickness of the NPLs and the ultra-high Pt SA loading. The surface-adsorbed Pt SAs show higher catalytic stability for photocatalytic hydrogen evolution than the substituted SAs, and this activity is higher when substitution is strictly excluded.

## Results

### Choice of model support

**Fig. 2:**
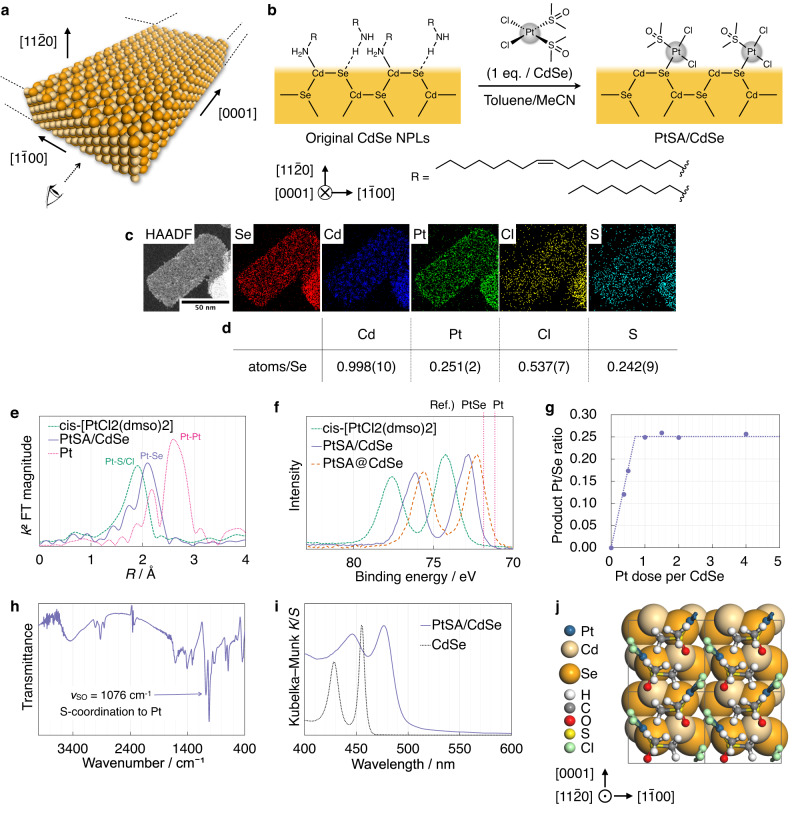
Surface adsorption of Pt SAs on CdSe NPLs. **a** Space-filling model of the 1.4-nm-thick wurtzite CdSe NPLs used in this study. **b** Schematic representation of the $(11{\bar{2}}0)$ surface structure of the CdSe NPLs and the Pt adsorption process from the direction indicated by the eye in (**a**). MeCN: acetonitrile. **c** High-angle annular dark-field (HAADF)–STEM image and the corresponding STEM–EDX elemental mapping of PtSA/CdSe. **d** Elemental composition of PtSA/CdSe determined using a combination of ICP–OES (Cd/Se, Pt/Se) and SEM–EDX (Cl/Cd, S/Cd). **e** *k*^2^-weighted Fourier-transform (FT) magnitude EXAFS spectra at the Pt L_3_ edge. **f** XPS Pt 4 *f* spectra of *cis*-[PtCl_2_(dmso)_2_], PtSA/CdSe, and PtSA@CdSe normalised at peak maxima. dmso: dimethyl sulfoxide. **g** Product Pt/Se ratios for different doses of the Pt precursor, determined by ICP–OES and SEM–EDX. **h** IR spectrum of PtSA/CdSe. **i** Vis–DR spectra normalised at peak maxima. **j** Surface structure model of PtSA/CdSe based on the XRD data for PtSA/CdSe and the reported crystal structure of *cis*-[PtCl_2_(dmso)_2_]. The unit cells are shown in grey lines. The CdSe and PtCl_2_(dmso) sections are shown in space-filling and ball-and-stick styles, respectively. Source data are provided as a Source Data file.

We chose 1.4-nm-thick wurtzite CdSe NPLs^32^ (Fig. 2a) as a model support material for location-selective Pt SA loading because their atomically defined thickness and surface structure^33,34^ enable a precise analysis of product structures and Pt SA location control, and because their high surface area allows a loading of Pt SAs that is sufficiently high for detailed characterisation.

The CdSe NPLs were synthesised according to a previously reported method^32^. The synthesised NPLs had lateral dimensions of 50–100 nm × 100–200 nm according to transmission electron microscopy (TEM) (Fig. S1). The uniform thickness of 1.4 nm was confirmed by the sharp excitonic peak at 453 nm in the ultraviolet–visible (UV–Vis) extinction spectrum, which is in good agreement with the reported value of 451 nm (Fig. S2). X-ray diffraction (XRD) analysis confirmed their crystal structure to be wurtzite, with ±$(11{\bar{2}}0)$ aligned to the thickness direction (Fig. S3). Infra-red (IR) spectroscopic analysis confirmed the presence of surface *n*-octylamine and oleylamine ligands (Fig. S4), which passivate the wide ±$(11{\bar{2}}0)$ facets through nitrogen coordination and hydrogen bonding to the surface Cd and Se, respectively, according to a previous study (Fig. 2b)^35^.

### Surface adsorption of Pt SAs

For surface-selective chemical adsorption of Pt SAs on the CdSe NPLs, the CdSe NPLs were mixed with 1 equivalent of *cis*-[PtCl_2_(dmso)_2_] per CdSe unit in toluene/MeCN solvent (Fig. 2b). The Pt SAs are adsorbed over the entire surface of each NPL, as confirmed by scanning TEM–energy-dispersive X-ray spectroscopy (STEM–EDX) (Fig. 2c). The product is hereafter denoted as PtSA/CdSe. High-angle annular dark-field STEM (HAADF–STEM) and TEM images of PtSA/CdSe confirm the absence of Pt aggregation (Figs. S5 and S6). Extended X-ray absorption fine structure (EXAFS) analysis shows no prominent peak for Pt–Pt bonding (Fig. 2e and S7). The Pt 4 *f* XPS spectrum shows the absence of metallic Pt (Fig. 2f). XRD measurement also shows the absence of crystalline Pt (Fig. S8). These results strongly indicate that Pt is distributed as SAs in PtSA/CdSe.

PtSA/CdSe is formed through an adsorption process rather than the substitution of Cd^2+^ with Pt^2+^. Inductively coupled plasma optical emission spectroscopy (ICP–OES) analysis reveals that as the dosing quantity of the Pt precursor increases, the Cd/Se atomic ratio is unchanged while the Pt/Se ratio plateaus at 0.25 (Fig. 2g). This substoichiometric saturation indicates that this adsorption process is surface-limited. Assuming the thickness of the NPLs to be 1.4 nm, they consist of 3.5 or 4 layers of CdSe^32^. If the NPLs contain four layers, half of the layers are exposed at the top and bottom surfaces. In those two layers, half of the Se atoms are exposed at the ridge of the corrugated structure (Fig. S9)^35^. Therefore, a quarter of the total Se atoms in the NPLs are exposed on the surface, which agrees with a maximum Pt/Se value of 0.25. In addition, the XPS spectrum of PtSA/CdSe shows Pt 4 *f* peaks at positions between those of the precursor *cis*-[PtCl_2_(dmso)_2_] and the substituted Pt SAs in PtSA@CdSe (*vide infra*) (Fig. 2f). These results indicate that Pt SAs in PtSA/CdSe are adsorbed on the surface without substitution of Cd.

Then, we investigate how the Pt SAs are adsorbed onto the CdSe NPL surface. The Fourier-transform (FT) EXAFS spectrum of PtSA/CdSe in Fig. 2e shows new Pt–Se bonding, indicating the adsorption of Pt species by coordination with surface Se. Although it is unclear whether Pt is coordinated with S or Cl because of the overlap of Pt–S, Cl, and Se peaks in EXAFS, the STEM–EDX mapping shows the uniform distribution of S and Cl along with Pt (Fig. 2c). The EXAFS spectrum was further analysed by curve-fitting to determine coordination numbers, but PtSA/CdSe were found to decompose during sample preparation, increasing Pt–Se and decreasing Pt–Cl coordination numbers (SI section “XAFS Analyses”). Therefore, only the upper/lower limits of coordination numbers are suggested: the coordination numbers of Pt–Se and Pt–Cl are less than 2.3(2) and more than 0.7(2), respectively. This result agrees with surface adsorption by one or two Pt–Se bonds, but partial contribution from substitutional incorporation at Cd sites with four Pt–Se bonds cannot be ruled out. To determine the coordination environment in detail, PtSA/CdSe was analysed by ICP–OES, scanning electron microscopy–EDX (SEM–EDX), and IR transmittance. SEM–EDX and ICP–OES analysis results in Fig. 2d reveal a Pt/S/Cl atomic ratio of 1:1:2, strongly indicating that only one dmso ligand is removed from *cis*-[PtCl_2_(dmso)_2_] during the adsorption process. The IR spectrum shows a single S–O stretching band at 1076 cm^−1^, indicating that the remaining dmso ligand is coordinated to Pt^2+^ through an S atom (Fig. 2h and S10 and Table S1). Therefore, it is concluded that the adsorption of Pt species proceeds via a ligand exchange reaction of dmso in *cis*-[PtCl_2_(dmso)_2_] with a surface Se of the CdSe NPLs, as depicted in Fig. 2b.

This adsorption process occurs with the desorption of the original amine ligands (*n*-octylamine and oleylamine) from the surface Cd and Se ions. The IR spectrum shows a decrease in the intensity of NH_2_ and CH_2_ stretching bands relative to CH_3_ stretching bands (Fig. S10), and the small-angle X-ray scattering (SAXS) results show a decrease in the stacking distances of NPLs from 3.4 to 1.8 nm, which indicates the replacement of long-chain *n*-octylamine and oleylamine ligands with short PtCl_2_(dmso) units (Fig. S11). Such desorption of amine ligands is caused by an attack of *cis*-[PtCl_2_(dmso)_2_] precursors. Before adsorption saturation, the quantity of Pt adsorbed increases linearly with the quantity of Pt dosed with a slope of 1/3 (Fig. 2g). This result indicates that 2/3 of *cis*-[PtCl_2_(dmso)_2_] is consumed in removing the amines by forming [PtCl_2_(amine)(dmso)] complexes (two amine molecules for every surface Cd–Se pair), and then the remaining 1/3 of the *cis*-[PtCl_2_(dmso)_2_] is adsorbed onto free surface Se after releasing one dmso ligand.

The CdSe NPL morphology is preserved in PtSA/CdSe, as shown by the TEM image in Fig. S6. Furthermore, the XRD measurements show similar patterns, indicating no significant change in CdSe crystal structure and crystallinity (Fig. S8). As a side note, the low-angle shift of the sharp peaks suggests the slight lattice expansion in the lateral directions of NPL presumably because of the steric clash between surface PtCl_2_(dmso) units (Table S2, Fig. 2j and S12). The visible diffuse reflectance (Vis–DR) spectrum still exhibits an excitonic absorption peak, indicating that thickness uniformity is more or less maintained (Fig. 2i).

Note that the absorption peak of the NPLs is red-shifted and broadened upon Pt adsorption. Similar changes of the UV–Vis absorption peak have been reported for CdCl_2_-passivated CdSe NPLs, and they are explained in terms of an increase in the effective thickness of the NPLs, which is caused by a combination of the electronic coupling between CdSe and surface species and the change of CdSe lattice constant induced by ligand exchange^36,37^.

Based on the above considerations, we construct a structural model of PtSA/CdSe, as shown in Fig. 2j. The lattice constants of the CdSe NPLs and the coordination geometry around Pt are taken from the XRD data for PtSA/CdSe and the single-crystal XRD structure of *cis*-[PtCl_2_(dmso)_2_]^38^, respectively. The model shows that PtCl_2_(dmso) units fill the CdSe $(11{\bar{2}}0)$ surface, providing ideal tetrahedral geometry to the surface Se (Fig. S12).

Among the precursors tested in our experiments, *cis*-[PtCl_2_(dmso)_2_] is optimal for Pt SA adsorption (Table S3). H_2_PtCl_6_, a typical precursor for loading Pt SAs^39^, is not suitable for this system. The reaction product with H_2_PtCl_6_ shows the formation of many holes in NPLs and the disappearance of the exciton peaks, confirmed by TEM and vis–DR spectroscopy, respectively (Figs. S13 and S14). It can be presumed that the oxidation of Se^2–^ by Pt^4+^ destroyed the CdSe NPL structure. Pt precursors with other ligands, such as *cis*-[PtCl_2_(NCPh)_2_] and [PtCl_4_]^2−^, attach less Pt to the CdSe NPLs, suggesting that the strong trans effect of dmso is necessary for adsorption via ligand exchange reactions.

### Internal substitution of Pt SAs

**Fig. 3:**
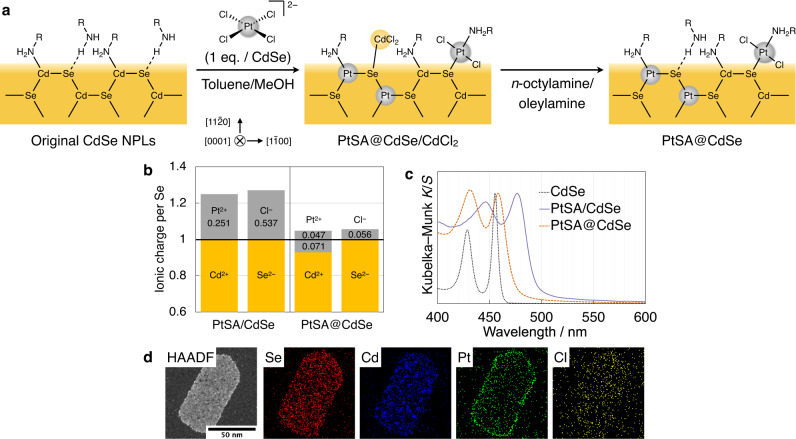
Internal substitution of Pt SAs into CdSe NPLs. **a** Schematic representation of the reaction process. MeOH: methanol. **b** Elemental compositions of PtSA/CdSe and PtSA@CdSe shown in ionic charge per Se as determined by ICP–OES and SEM–EDX. **c** Vis–DR spectra of the original CdSe NPLs, PtSA/CdSe and PtSA@CdSe normalised at the first exciton peak maxima. **d** High-angle annular dark-field (HAADF)–STEM image and the corresponding STEM–EDX elemental mapping of PtSA@CdSe. Source data are provided as a Source Data file.

Changes in reaction conditions switch the immobilisation of Pt species from adsorptive to substitutional incorporation within the interior of CdSe NPLs (Table 1). First, changing the solvent from toluene/MeCN (1:1, *v/v*) to toluene/MeCN/MeOH (2:1:1, *v/v/v*) causes a decrease in the Cd/Se ratio and an increase in the Pt/Se ratio, indicating that substitution of Cd^2+^ with Pt^2+^ occurs as well as adsorption in the presence of MeOH (entry 1 vs. 2). Changing the Pt precursor from *cis*-[PtCl_2_(dmso)_2_] to (CTA)_2_[PtCl_4_] (CTA = *n*-hexadecyltrimethylammonium) decreases the Pt/Se ratio, indicating suppression of adsorption by weaker trans effects (entry 3). However, the quantity of substituted Pt in this product cannot be estimated from the Cd/Se ratio because the free surface Se can bind CdCl_2_ generated by the substitution reaction (Fig. 3a)^37^. This surface CdCl_2_ can be successfully removed by treatment with a *n*-octylamine/oleylamine mixture, as confirmed by decreases in Cd/Se and Cl/Se ratios (entry 4). Increasing the reaction temperature from 20 °C to 30 °C further promotes the substitution reaction, as shown by the decrease in Cd/Se (entries 5, 6). Cd species (2.6% of total Cd) were detected in the supernatant of entry 5, further confirming the substitution reaction. The product of entry 6 is characterised in detail and is hereafter denoted PtSA@CdSe.

**Table 1:** Adsorption vs. substitution of Pt SAs on/in CdSe NPLs under different conditions

Entry	Solvent (v/v)	Reagent	Temp. (°C)	Workup	Cd/Se^a^	Pt/Se^a^	Cl/Se^a^	S/Se^a^
1	Toluene/MeCN (50:50)	[PtCl_2_(dmso)_2_] (1 eq)	20	None	0.998(10)	0.251(3)	0.537(7)	0.242(9)
2	Toluene/MeCN/MeOH (50:25:25)	[PtCl_2_(dmso)_2_] (1 eq)	20	None	0.973(10)	0.270(3)	0.515(5)	0.242(2)
3	Toluene/MeOH (50:50)	[PtCl_4_]^2−^ (1 eq)	20	None	0.967(3)	0.103(1)	0.155(2)	N.D.
4	Toluene/MeOH (50:50)	[PtCl_4_]^2−^ (1 eq)	30	None	0.974(10)	0.175(2)	0.292(3)	N.D.
5	Toluene/MeOH (50:50)	[PtCl_4_]^2−^(1 eq)	20	Amines	0.955(3)	0.070(1)	0.065(2)	N.D.
6	Toluene/MeOH (50:50)	[PtCl_4_]^2−^ (1 eq)	30	Amines	0.929(9)	0.118(1)	0.112(3)	N.D.
7	Toluene/MeOH/H_2_O (49:49:2.5)	[PtCl_4_]^2−^ (0.5 eq)	30	Amines	0.939(9)	0.148(1)	0.140(2)	N.D.
8	Toluene/MeOH/AcOH (50:50 + 2 eq.)	[PtCl_4_]^2−^ (0.5 eq)	30	Amines	0.880(9)	0.144(1)	0.123(7)	N.D.
9	Adsorption-substitution hybrid loading (see the text)				0.951(10)	0.303(3)	0.522(10)	0.205(11)

*N.D.* not detected

^a^Atomic ratio determined by a combination of ICP–OES (Cd/Se, Pt/Se) and SEM–EDX (Cl/Cd, S/Cd).

The quantities of adsorbed vs. substitutional Pt SAs are visualised in Fig. 3b. In contrast to the fully adsorptive deposition of Pt SAs (0.251 per Se) in PtSA/CdSe, PtSA@CdSe contains a major contribution from Cd substitution (0.071 per Se) along with a minor contribution from adsorption (0.047 per Se). The presence of Cl (0.056 per Se in ionic charge) suggests that the minor adsorption species is PtCl_2_.

As observed in PtSA/CdSe, Pt species are incorporated as SAs in PtSA@CdSe, as evidenced by the lack of Pt–Pt bonding in the EXAFS spectrum (Figs. S15 and S45), the lack of reflections for crystalline Pt in XRD (Fig. S16), the absence of Pt clusters in HAADF–STEM (Fig. S17), and the absence of metallic Pt in XPS (Fig. 2f).

However, the coordination environment of Pt in PtSA@CdSe is markedly different from that in PtSA/CdSe. The Pt 4*f* XPS spectra in Fig. 2f show that the Pt in PtSA@CdSe has a lower binding energy closer to the reported value of PtSe^40^ than the Pt in PtSA/CdSe, suggesting that more Se atoms are coordinated to the Pt in PtSA@CdSe because of substitution.

The Pt distribution in a PtSA@CdSe NPL is compared with that in PtSA/CdSe by means of HAADF–STEM images and STEM–EDX maps, showing that Pt is more abundant at the periphery than at the centre of the NPLs, while Cl is uniformly distributed over the NPLs (Fig. 3d and S18). This result strongly suggests that the substitution reaction proceeds from the NPL side faces, while the PtCl_2_ species are uniformly adsorbed over the top and bottom faces of the NPLs, as observed for PtSA/CdSe. This anisotropic reactivity is reasonable because the side faces of NPLs are less protected by amine ligands and more reactive than the top and bottom faces, which is the basis of the anisotropic growth of CdSe NPLs.

As previously observed in cation exchange reactions of ionic nanocrystals^41^, the morphology of the CdSe NPLs is maintained during the substitution reaction, as observed by TEM (Fig. S19). XRD measurements also confirm the maintained CdSe crystal structure and no formation of PtSe crystallites (Fig. S16). The IR spectrum is almost identical to that of the pristine CdSe NPLs, indicating a preserved surface structure (Fig. S20). In the Vis–DR spectrum shown in Fig. 3c, the PtSA@CdSe still exhibits an excitonic absorption peak, indicating that the uniformity of thickness is maintained after the substitution reaction (Fig. 3c). In contrast to PtSA/CdSe, only a slight red-shift of the absorption peak from that of the original CdSe NPLs is observed, indicating the small quantity of adsorbed Pt species.

We also investigate what promotes the substitution process. The addition of a small amount of H_2_O causes faster substitution, providing a similar amount of substituted Pt even with half the amount of [PtCl_4_]^2−^ (Table 1, entry 7). Furthermore, adding a tiny amount of acetic acid induces a much faster substitution reaction (entry 8). From these results, it can be inferred that the acidic protons interact with surface Se or amine ligands by hydrogen bonding, weakening their bonds with Cd^2+^ ions, and facilitating the removal of Cd^2+^ from the lattices.

These results show that the formation mechanisms of PtSA/CdSe and PtSA@CdSe are contrastive, where (1) PtSA/CdSe is formed via a simple ligand exchange process of Pt complexes, while the PtSA@CdSe is formed via a cation exchange reaction of CdSe with Pt^2+^; (2) the formation of PtSA/CdSe is promoted by the trans effect of ligands in the Pt complexes, while the formation of PtSA@CdSe is promoted by acidic protons; and (3) the adsorption of PtSAs on CdSe occurs on the top and bottom faces of the NPLs, while the substitution of Cd^2+^ with Pt^2+^ in CdSe proceeds from the side faces of the NPLs. Thus, coordination chemistry can be used to control the location of PtSAs on the surface or within the interior of CdSe NPLs.

Since the Pt content of PtSA@CdSe (Pt/Se 0.118(1)) was much lower than that of PtSA/CdSe (Pt/Se 0.251(3)), we also prepared samples with increased Pt contents. When CdSe NPLs are treated with a larger amount of (CTA)_2_[PtCl_4_] or a longer reaction time, an increase in Pt/Se atomic ratio and a decrease in Cd/Se were observed by ICP–OES and SEM-EDX, indicating further incorporation of substitutional Pt SAs (Table S4). However, TEM images showed the formation of dark particulate domains (Fig. S21). The valence of Pt calculated from Cd, Pt, Se and Cl atomic ratios is +2, ruling out the reduction of Pt^2+^ to metallic Pt nanoparticles (NPs) (Table S4). Therefore, the newly formed particles are most likely PtSe species. This result indicates that a high loading of substitutional Pt SAs causes the segregation of PtSe phases. Therefore, PtSA@CdSe (Pt/Se 0.118(1)) is the highest Pt content achieved without phase segregation.

### Hybrid adsorption–substitution loading of Pt SAs

To confirm whether the adsorption and substitution proceed independently, we examine the stepwise reaction of these two processes. CdSe NPLs were subjected first to the substitution reaction, followed by an adsorption reaction (Fig. S22). The product is hereafter denoted PtSA@CdSe/PtSA.

This stepwise reaction does not significantly change the original morphology of the CdSe NPLs nor induce aggregation of Pt SAs, as confirmed by TEM (Fig. S23). ICP–OES analysis reveals a loss of Cd caused by the substitution, while the Pt/Se ratio (0.303(3)) is in accordance with the sum of the saturated adsorption (0.250) and the loss of Cd (1 – 0.951(10) = 0.049(10)) (Table 1, entry 9). The IR spectrum is similar to that of PtSA/CdSe (Fig. S24). These results demonstrate that the adsorption and substitution processes can independently introduce different types of Pt SAs to CdSe NPLs.

### Effects of Pt SA location on the electronic structure of the product

**Fig. 4:**
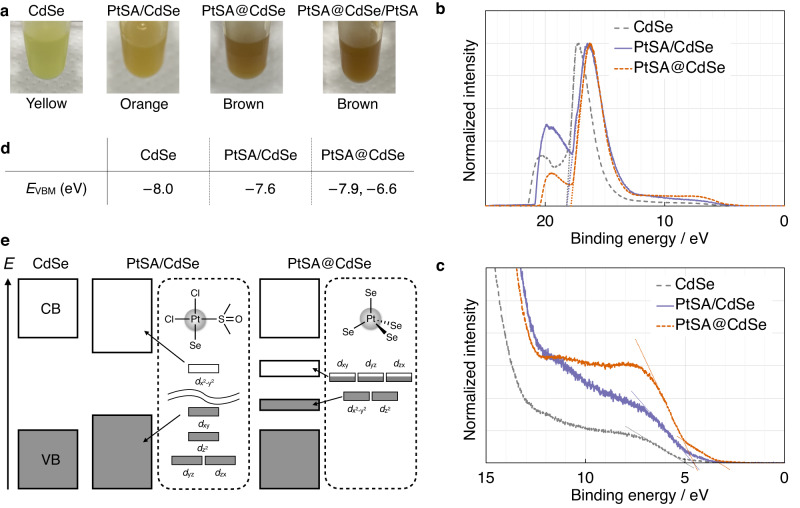
Electronic structure investigation of products. **a** Photographs showing the colours of the original CdSe NPLs and the three products. **b** UPS spectra of the original CdSe NPLs and the products, normalised at the peak maxima. The dotted straight lines show the extrapolation of the secondary electron cut-off feature used to obtain *E*_high_ values. Sub-peaks due to sample charging were ignored. **c** Magnified view of (**b**). The dotted straight lines show the extrapolation of the valence band maximum cut-off used to obtain *E*_low_ values. **d** Valence band maximum energies (*E*_VBM_) vs. vacuum calculated from the UPS spectra. **e** Plausible band diagrams of the original CdSe NPLs and the products, with the crystal-field splitting patterns of the Pt *d* orbitals. CB conduction band, VB valence band. Source data are provided as a Source Data file.

The PtSA/CdSe has an orange colour similar to that of pristine CdSe NPLs, whereas PtSA@CdSe and PtSA@CdSe/PtSA exhibit darker brown colours, suggesting the formation of midgap levels in the bandgap of CdSe NPLs upon the incorporation of Pt SAs (Fig. 4a)^42^. Regrettably, midgap levels cannot be confirmed from the Vis–DR spectrum owing to overlap with the tails of excitonic absorption (Fig. S25). Therefore, we conducted ultraviolet photoelectron spectroscopy (UPS) measurements (Fig. 4b, c). Fig. 4d shows the valence band maximum energies (*E*_VBM_) vs. vacuum calculated by the following equation:

1 $${E}_{{{{{{\rm{VBM}}}}}}}=-h\nu+({E}_{{{{{{\rm{high}}}}}}}-{E}_{{{{{{\rm{low}}}}}}})$$

where *hν* is the excitation energy (21.2 eV), and *E*_high_ and *E*_low_ are the high and low cut-off energies of the UPS spectrum, respectively. The *E*_VBM_ of the pristine CdSe NPLs is calculated to be –8.0 eV, which agrees with the value reported for 4.0 nm CdSe nanocrystals measured by UPS (–7.60 eV)^43^.

*E*_VBM_ of PtSA/CdSe is slightly shifted to higher energy (–7.60 eV) with respect to the pristine CdSe NPLs. The adsorbed Pt SAs in PtSA/CdSe should take square-planar geometry, which is most favourable for Pt^2+^ and induces large crystal-field splitting of *d* orbitals. Thus, these occupied and unoccupied orbitals can only participate in the valence band and conduction band of CdSe NPLs, resulting in a small increase of *E*_VBM_ (Fig. 4e). The main edge feature of PtSA@CdSe corresponds to *E*_VBM_ = –7.9 eV, which is closer to that of pristine CdSe than to that of PtSA/CdSe and indicates the scarcity of the adsorbed Pt SAs.

Interestingly, the UPS spectrum of PtSA@CdSe shows a more intense signal than those of other samples at binding energies lower than 12 eV and an additional small edge slope that corresponds to *E*_VBM_ = –6.6 eV. This indicates the formation of midgap states in the bandgap of CdSe, which is a similar situation to that observed for Cu-doped CdSe nanocrystals^43,44^. These midgap levels could be attributed to the different coordination geometry of Pt SAs and crystal-field splitting of *d* orbitals (Fig. 4e). The substitutional Pt SAs in PtSA@CdSe need to adopt a tetrahedral geometry like that of Cd^2+^ in CdSe, resulting in the weak crystal-field splitting of *d* orbitals within the bandgap.

### Photocatalytic hydrogen evolution over CdSe NPLs with Pt SAs in different locations

In general, cadmium chalcogenide NPLs are powerful photocatalysts owing to their strong light absorption ability and short mean exciton diffusion length to the surface^45–47^. Their photocatalytic performances can be dramatically enhanced by loading with Pt nanoparticles^48–50^. Pt SAs can serve in the same manner, as demonstrated in the case of CdS nanowires^39^. Pt species work both as an electron acceptor and as a catalyst, assisting both charge separation and hydrogen evolution^39,49^.

**Fig. 5:**
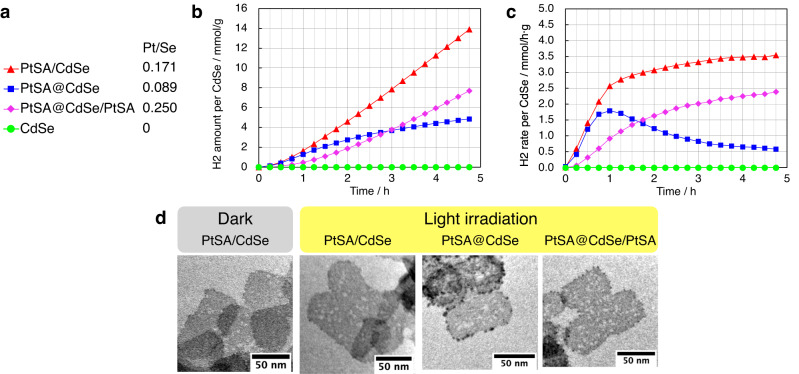
Photocatalytic hydrogen evolution over CdSe NPLs with different Pt SA locations. **a** Legend for the graphs and Pt/Se atomic ratios determined by ICP–OES. **b** Total evolved H_2_ amount per original CdSe under visible-light irradiation (Xe lamp with a > 422 nm filter, 1000 W/m^2^) in H_2_O/TEOA (4:1, *v/v*) at 25 °C. **c** Change in H_2_ evolution rate over time in the same conditions. **d** TEM images of catalysts recovered after catalysis. For PtSA/CdSe, a control sample without light irradiation is also shown. Source data are provided as a Source Data file.

We investigate the influence of Pt SA location on/in CdSe NPLs on photocatalytic hydrogen evolution performance. Before photocatalysis, we conducted ligand exchange with a hydrophilic 11-mercpatoundecanoic acid (MUA) to make the NPLs water-dispersible. We employed a typical one-step exchange method^51^ for PtSA/CdSe (Fig. S26). Complete ligand exchange from dmso and Cl to deprotonated MUA is confirmed by IR and SEM–EDX (Fig. S27 and Table S5). The product can be dispersed well in water, as confirmed by the weak scattering at the long-wavelength region in the UV–vis extinction spectrum (Fig. S37). The morphology and crystal structure of catalysts are maintained, as confirmed by TEM and XRD (Figs. S28 and S29). Although a partial loss of Pt during ligand exchange is confirmed by ICP–OES (Pt/Se from 0.251 to 0.171), the Cd/Se atomic ratio is maintained (Table S5). The coordination environment around Pt is slightly changed by the exchange of Cl with MUA, as confirmed by EXAFS (Fig. S30). A similar result was obtained for PtSA@CdSe/PtSA (Figs. S27 and S28 and Table S5). Because the ligand exchange with MUA works only when all the original amine ligands on CdSe are replaced with PtCl_2_(dmso), the Pt contents of the catalysts are fixed (Fig. 5a).

For PtSA@CdSe and the pristine CdSe, however, the direct ligand exchange from amines to MUA is impossible because of the strong binding of amine ligands^52^. Therefore, the NPLs were treated first with CdBr_2_ to replace surface amines^36^. Then, deprotonated MUA was introduced by replacing the Br^−^ of the surface CdBr_2_, yielding MUA-coated PtSA@CdSe and CdSe NPLs (Fig. S31). Complete ligand exchange is confirmed by IR and SEM–EDX (Fig. S32 and Table S6). The products are dispersible in water while keeping their original morphology, as confirmed by TEM (Fig. S33). The quantity of Pt in PtSA@CdSe slightly decreases during this two-step ligand exchange process owing to the loss of adsorbed Pt SAs (Table S6).

Although it would be ideal to study the effect of Pt loading on the catalytic activity and stability, it is technically challenging to regulate Pt loading in our catalytic setup. Although the loading of surface-adsorbed Pt SAs before ligand exchange can be tuned by the amount of *cis*-[PtCl_2_(dmso)_2_] (Fig. 2g), the use of excess *cis*-[PtCl_2_(dmso) _2_] is necessary for aqueous dispersion. This is because the ligand exchange with MUA is effective only when all the original amine ligands on CdSe are replaced with PtCl_2_(dmso). If the dispersion state of the catalyst could be controlled by other methods, catalytic performance could be maximised by optimising Pt loading, as observed in other Pt SA catalysts^24,30^. The Pt loading in PtSA@CdSe is Pt/Se = 0.089 (after ligand exchange) at most and cannot be increased due to the aforementioned PtSe segregation at higher loading. Therefore, the catalytic performances of PtSA/CdSe and PtSA@CdSe are compared while considering this difference in Pt loading.

Using these water-dispersible NPLs, photocatalysis was conducted under visible-light irradiation ( > 422 nm) in the presence of triethanolamine (TEOA) as a sacrificial electron donor. Pt SA-loaded CdSe NPLs show photocatalytic activity for the hydrogen evolution reaction while pristine CdSe NPLs show no activity in this system (Fig. 5a, b). PtSA/CdSe shows a stable activity of 3.5 mmol h^−1^ g^−1^ for 5 h, which is comparable to state-of-the-art Pt/CdSe systems without heterojunctions (Table S8). On the other hand, PtSA@CdSe exhibits a gradual decrease in activity over time (Fig. 5c). This instability makes PtSA@CdSe a poorer catalyst than PtSe/CdSe in total evolved amounts of H_2_ after 2.5 h, whether they are normalised by the mass of CdSe or Pt (Fig. S34). PtSA@CdSe/PtSA has a lower activity than PtSA/CdSe although it contains a larger quantity of Pt (Fig. 5a).

PtSA/CdSe mostly preserves its nanostructure, composition and dispersion state after photocatalysis for 5 h, as confirmed by TEM, ICP–OES, SEM–EDX, IR, and UV–Vis extinction spectra (Fig. 5d, S35–S37 and Table S7). Although TEM images show the formation of some holes (Fig. 5d and S35), this is presumably due to aerobic oxidation during extensive ultracentrifugation required for catalyst collection. The UV–Vis extinction spectra of the catalyst dispersion before and after photocatalysis without the catalyst collection process show a negligible change (Fig. S37), and a control experiment without light irradiation gives similar hole formation (Fig. 5d and S38). The TEM image (Fig. 5d) also show small dark particles, suggesting the formation of Pt NPs. However, the UV–Vis extinction spectrum (Fig. S37) does not exhibit significant absorption or scattering from Pt NPs, and the ICP–OES/SEM–EDX analyses show a S/Pt atomic ratio of 1.938(12) after photocatalysis, indicating that the Pt2+ state coordinated with two deprotonated MUA ligands is mostly maintained.

In contrast, many dark particulate domains are observed on the NPL edges in the TEM images of PtSA@CdSe after photocatalysis (Fig. 5d and S35). The decomposition of the PtSA@CdSe structure is also confirmed by the decrease in absorbance and broadening in the UV–Vis spectrum (Fig. S37). The formation of metallic Pt NPs is dismissed by the ICP–OES data, which show unchanged Pt/Se atomic ratio (0.0895(9) → 0.0910(9)) throughout photocatalysis since the reduction of Pt–Se species to Pt should increase the Pt/Se ratio. Therefore, the decomposition is most likely the segregation of the PtSe phase. The segregation would be driven by the strained coordination geometry of substituted Pt SAs, which need to take a tetrahedral geometry to adapt to the CdSe lattice, as supported by the aforementioned UPS analysis, although Pt^2+^ favours a square-planar geometry. This decomposition may be responsible for the low catalytic stability for hydrogen evolution (Fig. 5c). The decomposition via the segregation of PtSe phases is also observed in the aforementioned experiments to increase the Pt loading of PtSA@CdSe. This result indicates that the poorer catalytic stability of PtSA@CdSe than PtSA/CdSe is not due to the lower Pt loading, since a higher Pt loading leads to much lower stability. Therefore, the poorer catalytic stability of PtSA@CdSe is attributed to the difference in the location of PtSAs

To further investigate the long-term stability of PtSA/CdSe, we conducted longer photocatalysis experiments for 20 h and 100 h. In all experiments, the hydrogen evolution rate was constant at approximately 3.5 mmol h^−1^ g^−1^ for the initial 20 h, and then slightly increased to 4.5 mmol h^−1^ g^−1^ over the subsequent 60 h, indicating that the significant structural transformation took place after 20 h (Fig. S39). The UV–vis extinction spectra showed negligible changes before and after photocatalysis for 5 h and 20 h, while the 100 h run exhibited a significant change (Fig. S40). The TEM images of the recovered catalysts revealed a gradual increase in the number and size of small dark particles for longer photocatalysis, especially prominent after 100 h (Fig. S41). These results indicate that the PtSA/CdSe underwent a significant structural transformation after 20 h of photocatalysis. Because the segregation of PtSe similar to the case of PtSA@CdSe cannot explain the increase in activity, we inferred that adsorbed Pt SAs were gradually reduced to Pt NPs (dark spots in TEM images) to serve as an active co-catalyst for photocatalytic hydrogen evolution reaction^53^. The loss of surface-adsorbed Pt SAs caused a blue-shift of exciton transitions (Fig. 2i), and the absorption and scattering by the formed Pt NPs caused an increase in extinction intensity (Fig. S40c). The loss of adsorbed Pt SAs also makes the CdSe surface susceptible to aerobic oxidation during sample handling for TEM observation, which can explain the extensive hole formation in TEM image (Fig. S41d). The prominent transformation after 20 h could be triggered either by the depletion of the surface ligand MUA due to oxidation or by the interaction with the accumulated decomposition products of the sacrificial electron donor TEOA^54^. Nonetheless, the PtSA/CdSe structure was almost maintained for 20 h, as confirmed by the steady catalytic activity and unchanged UV–vis extinction spectra, in contrast to PtSA@CdSe, which decomposed within 1 h under the same conditions. The adsorbed Pt SAs should be responsible for the initial 20 h catalytic activity (3.5 mmol h^−1^ g^−1^) because the Pt SAs are more abundant and more positively charged to attract excited electrons for hydrogen evolution in comparison with the Pt NPs. It is noteworthy that the activities of both initial PtSA/CdSe (3.5 mmol h^−1^ g^−1^) and transformed PtNP/CdSe (4.5 mmol h^−1^ g^−1^) are comparable to state-of-the-art Pt/CdSe systems without heterojunctions (Table S8), while the PtNP/CdSe is quite stable for over 80 h.

These results demonstrate that the stability of PtSA–CdSe systems in photocatalytic hydrogen evolution reaction strongly depends on the location of Pt SAs. When Pt SAs are adsorbed on the surface of CdSe NPLs, the system is mostly stable and active for photocatalytic hydrogen evolution for 20 h under our conditions. Surface-adsorbed Pt SAs are gradually reduced into Pt NPs after 20 h under our conditions to give higher activity for hydrogen evolution. On the other hand, when Pt SAs are substitutionally incorporated within the interior of CdSe NPLs, the quick phase segregation of PtSe occurs within 1 h to decrease the catalytic activity under the same conditions.

The higher stability of PtSA/CdSe than PtSA@CdSe can stem from several factors. First, as discussed above, the strained coordination geometry of Pt^2+^ in PtSA@CdSe can drive phase segregation. Second, the Pt SAs in PtSA/CdSe is coordinated with deprotonated MUA during photocatalysis, as confirmed by IR and SEM-EDX before and after catalysis (Fig. S36 and Table S7). The presence of such a strong ligand can stabilise the Pt SAs. Third, in photocatalysis, photogenerated electrons are first trapped by Pt SAs under photoirradiation^39^. The trapped electron in PtSA@CdSe can destabilise the lattice, while in PtSA/CdSe the trapped electron resides on the surface without affecting the lattice. This mechanism can also explain why PtSA@CdSe/PtSA is more stable than PtSA@CdSe.

The lower activity of PtSA@CdSe/PtSA than that of PtSA/CdSe is an unexpected result, considering the higher amount of Pt SAs in PtSA@CdSe/PtSA. This result indicates that the midgap levels formed by substituted Pt SAs, as shown in the UPS analysis (Fig. 4), inhibit photocatalysis by acting as recombination centres and/or trapping electrons at an energetically lower level^43^.

## Discussion

This research demonstrates that the surface vs. interior loading of Pt SAs on/in CdSe NPLs can be divergently controlled by coordination chemistry, specifically through judicious choice of the Pt precursor complex, solvent, and additional ligands. Such techniques will aid the synthesis of SA species on various supports with well-defined atomic distributions. The controlled distribution of SA species would be useful in research not only on their photocatalysis but also on their electrocatalysis^14,55^, thermocatalysis^30^, sensing^56^, and luminescence^57^.

Furthermore, surface Pt SAs on CdSe NPLs are effective for photocatalytic hydrogen evolution, while internal Pt SAs are rather detrimental to catalytic activity and stability owing to their unusual coordination environment. This finding indicates that precise location control is necessary for single-atom catalysts in order to elucidate their properties and obtain maximum catalytic activity and stability. This insight can be useful for SAs on other metal chalcogenides^24^, pnictides, and halides^16,58^ to improve their photocatalytic performance.

## Methods

### Chemicals

Cadmium chloride (CdCl_2_, 95%, FUJIFILM Wako), oleylamine (OAM, 70%, Sigma–Aldrich), *n*-octylamine (^*n*^OctNH_2_, 98.0%, FUJIFILM Wako), selenium (Se, 100 mesh, 99.99%, Sigma–Aldrich), tri-*n*-butylphosphine (TBP, 95.0%, TCI), ethanol (EtOH, 99.5%, Japan Alcohol Trading), *n*-hexane (95.0%, FUJIFILM Wako), toluene (99.0%, FUJIFILM Wako), methanol (MeOH, 99.5%, FUJIFILM Wako), chloroform (CHCl_3_, 99.0%, FUJIFILM Wako), activated basic alumina (Al_2_O_3_, for column chromatography, ca. 75 µm, pH 9.0–11.0 at 100 g/L, Wako), *cis*-dichlorobis(dimethyl sulfoxide)platinum(II) (*cis*-[PtCl_2_(dmso)_2_], 97%, Sigma–Aldrich), anhydrous acetonitrile (MeCN, 99.8%, super dehydrated, FUJIFILM Wako), *cis*-bis(benzonitrile)dichloroplatinum(II) (*cis*-[PtCl_2_(NCPh)_2_], 98%, Sigma–Aldrich), *cis*-dichlorobis(triphenylphosphine)platinum(II) (*cis*-[PtCl_2_(PPh_3_)_2_], 8.6–9.3% Cl, Sigma–Aldrich), hexadecyltrimethylammonium chloride (CTAC, 98.0%, Sigma–Aldrich) potassium tetrachloroplatinate(II) (K_2_PtCl_4_, 98.0% Wako), dichloromethane (DCM, 99.0%, FUJIFILM Wako), anhydrous methanol (MeOH, 99.8%, super dehydrated, FUJIFILM Wako), acetic acid (AcOH, 99.7%, Wako), potassium hydroxide (KOH, 85.0%, FUJIFILM Wako), 11-mercaptoundecanoic acid (MUA, 95%, Sigma–Aldrich), cadmium bromide tetrahydrate (CdBr_2_·4H_2_O, 98%, FUJIFILM Wako), dimethyl sulfoxide (DMSO, 99.8%, Kishida), triethanolamine (TEOA, 99.0%, Sigma–Aldrich), hydrochloric acid (HCl aq, 35.0%–37.0%, for atomic absorption spectrometry, Wako), nitric acid (HNO_3_ aq, 60%–61%, for metal analysis, Wako), and boron nitride (spectral grade, Wako) were used as received without further purification. Water was supplied using a Milli-Q system.

### Characterisation

Inductively coupled plasma optical emission spectroscopy (ICP–OES) data were recorded on an ICPE-9820 plasma atomic emission spectrometer (Shimadzu). For the untreated CdSe NPLs, the solids were collected by centrifugation, dried *in vacuo*, digested with fresh aqua regia at RT in a polypropylene tube, and diluted with water to 20 times its volume. Other samples were put into Teflon-lined autoclaves in a dispersion state and dried in air. Fresh aqua regia made of HCl aq and HNO_3_ aq (3:1 v/v) was added to the autoclave, and the autoclave was immediately sealed and heated at 150 °C for 4 h in a muffle furnace to digest the sample. The digested solution was diluted with water to 20 times its volume and subjected to measurement. Each sample was measured three times, and the values obtained were averaged. For each set of measurements, untreated CdSe NPLs were digested in an autoclave in parallel to correct the loss of cadmium by adsorption to the Teflon lining. The overall standard uncertainty was estimated to be ± 1%, mainly due to uncertainty in standard solution preparation.

Transmission electron microscopy (TEM) observations were performed on an HT7820 microscope (Hitachi High-Tech) at an accelerating voltage of 120 kV. The dispersion samples were drop-casted onto a carbon membrane supported by a Cu grid, dried *in vacuo*, and subjected to measurement. The samples after ligand exchange with MUA were dispersed in MeOH for drop-casting. For the samples after photocatalysis, the solvent was exchanged to MeOH by centrifugation before drop-casting.

High-angle annular dark-field scanning TEM (HAADF–STEM) and energy-dispersive X-ray spectroscopy (EDX) elemental mapping images were collected using a JEM-ARM200F microscope (JEOL) at an accelerating voltage of 80 kV. The dispersion samples were drop-casted onto a thin carbon membrane supported by a Triafol microgrid and Cu grid, dried *in vacuo*, and subjected to measurement.

Quantitative scanning electron microscopy (SEM)—EDX data were recorded on an S-4800 scanning electron microscope (Hitachi) equipped with an EDAX Apollo XF detector (Ametek) at an accelerating voltage of 30 kV. The powder samples were put onto a conductive carbon tape and subjected to measurement. Spectrum integration was conducted in three areas, each filled with tens of nanoplatelets. Standard uncertainty was estimated by the sample standard deviation of the three measurements. The S/Cd and Cl/Cd atomic ratios obtained from these measurements were combined with the Cd/Se atomic ratios obtained by ICP–OES to calculate the S/Se and Cl/Se atomic ratios. The direct quantification of the S/Se and Cl/Se atomic ratios by SEM–EDX was avoided because the larger difference of characteristic X-ray energies between S K, Cl K, and Se K than those with Cd L makes correction of the absorption effect inaccurate.

X-ray diffraction (XRD) patterns were obtained on an X’Pert Pro MPD powder diffractometer (PANalytical) with CuKα radiation (λ = 1.542 Å) and at the BL02B2 beamline in the SPring-8 facility of the Japan Synchrotron Radiation Research Institute (JASRI) with monochromatic X-rays (λ = 0.527536(1) Å). For CuKα radiation, the powder samples were put onto a zero-diffraction plate and subjected to measurement in Bragg–Brentano geometry. For synchrotron radiation, the powder samples were ground, put into a Lindemann glass capillary, and subjected to measurement in Debye–Scherrer geometry.

Small-angle X-ray scattering (SAXS) patterns were measured on a Nano-Viewer (Rigaku) instrument with CuKα radiation (λ = 1.542 Å) operated at 40 kV and 30 mA. The powder samples were put into a Kapton-film bag and subjected to measurement.

UV–Vis extinction spectra were measured on a U-3310 spectrophotometer (Hitachi). Dispersion samples were put into a 1-cm quartz cell and subjected to measurement.

UV–Vis diffuse reflectance (DR) spectra were obtained using a UH5700 spectrophotometer (Hitachi) with a BaSO_4_ plate as a reference. The dispersion samples were put into a 1-mm quartz cell, attached to an integrating sphere with a BaSO_4_ plate as the background, and subjected to measurement.

Infra-red (IR) spectra were recorded on an FT/IR-6600 Fourier-transform IR spectrometer (JASCO) at a resolution of 1 cm^–1^. The powder samples were pelletised using a TabletMaster system (JASCO) and KBr plates and subjected to measurements.

X-ray absorption fine structure (XAFS) measurements were conducted at the BL01B1 beamline in the SPring-8 facility of the JASRI. The incident X-ray beam was monochromatized with a Si(111) double-crystal monochromator. The X-ray energy was calibrated using Pt foil. The powder samples were ground with boron nitride, pressed into a pellet, and mounted on a metal holder attached to a cryostat. The spectra were measured in transmission mode with ionisation chambers. The data were processed using the Athena programme in the Demeter v0.9.26 package (Bruce Ravel) and Ifeffit v.1.2.12 (University of Chicago)^59^. The absorption edge energy was determined by the Ifeffit default method. Normalisation was conducted using a pre-edge range of −150 to −30 eV and a post-edge range of 150 to 1027 eV relative to the edge with a normalisation order of 3. The baseline was determined using the Autobk algorithm^60^ (*R*_bkg_ = 0.95, *k*-weight = 2, spline range in *k* = 0–16.4 Å^−1^, spline clamps: low, none; high, strong). The *k*^2^-weighted *χ*(*k*) data within the *k* range 3.0–15.9 Å^−1^ were Fourier-transformed into *R* space with a Hanning window function (d*k* = 1 Å^−1^). The quantitative curve-fitting was performed using an Artemis programme of the Demeter package. The functions of effective curved-wave backscattering amplitudes and phase shifts were calculated by an ab initio code FEFF v6L.02^61^. The fitting was conducted for the *R* range of 1.2–2.6 Å with a Hanning window function (d*R* = 0) with multiple *k*-weights of 1, 2, and 3. The coordination numbers (*N*), the interatomic distances (*R*), the Debye–Waller factors (*σ*^2^), and the edge energy shift (Δ*E*_0_) were allowed to change during the fitting unless otherwise noted.

X-ray photoelectron spectroscopy (XPS) and ultraviolet photoelectron spectroscopy (UPS) measurements were carried out with a Kratos AXIS Ultra-DLD ultra-high vacuum surface analysis system with a base pressure of 1 × 10^−7^ Pa. The powder samples were pressed onto an indium foil and subjected to measurement. XPS measurements were conducted with a monochromatic Al Kα source (1486.6 eV). The photoelectron emission angle to surface normal, θ, was set to 0°. The pass energy of the analyser and the energy step were 160 and 1.0 eV for wide scans, respectively, and 20 and 0.1 eV for narrow scans, respectively. A neutraliser partially compensated the charging of the sample surfaces. The binding energies of the XPS spectra were calibrated so that the binding energy of the C 1s peak of coordinated dmso ligands was 285.4 eV for PtSA/CdSe and *cis*-[PtCl_2_(dmso)_2_] in analogy to [Me_3_SO]^+^^62^, and the C 1s peak of the alkyl chains of the amine ligands was 285.1 eV for PtSA@CdSe^63^. UPS analysis was performed with an unfiltered He I (21.2 eV) lamp and a total instrumental energy resolution of 0.01 eV. The Fermi level cut-off of an Ag surface cleaned with an Ar ion beam was used to calibrate the energy scale.

### Synthesis

#### Synthesis of 1.4-nm-thick wurtzite CdSe ±(11$\bar{2}$0) nanoplatelets (CdSe NPLs)

The synthesis was performed based on a published procedure^32^ with some modifications. CdCl_2_ (3 mmol), ^*n*^OctNH_2_ (10 mL), and OAM (10 mL) were combined in a three-necked round-bottom flask connected to a Schlenk line. The inner atmosphere was replaced with nitrogen. The mixture was heated at 120 °C for 2 h and then cooled to RT. A mixture of Se (9 mmol), ^*n*^OctNH_2_ (5 mL), and OAM (5 mL) was added to the flask. The inner atmosphere was replaced with nitrogen. The temperature of the mixture was increased to 100 °C at 2 °C/min. The mixture was maintained at 100 °C for 16 h and then cooled to RT with a water bath. TBP (13.4 mL) was injected, and the mixture was sonicated to dissolve all grey powder. EtOH (50 mL) was added to the mixture, and the precipitate was collected by centrifugation and purified by three redispersion–centrifugation cycles in EtOH (93 mL), *n*-hexane/EtOH (1:1, 93 mL), and then toluene/MeOH (1:1, 93 mL) for each cycle. The precipitate was dispersed in CHCl_3_ (45 mL, percolated through activated basic Al_2_O_3_) and sonicated for 10 min to fully exfoliate the NPLs. The dispersion was concentrated to 10 mL using a rotary evaporator. The dispersion was diluted with toluene (10 mL) and *n*-hexane (80 mL) and centrifuged. The precipitate was redispersed in toluene (100 mL) and centrifuged. The final precipitate was redispersed in toluene (80 mL) for further use. The concentration of the CdSe formula unit in the dispersion was determined by ICP–OES for each batch.

#### Synthesis of PtSA/CdSe

A toluene dispersion of CdSe NPLs (37.9 mM in CdSe units, 79.2 µL), toluene (171 µL), and anhydrous MeCN (250 µL) were combined in a 2 mL microtube. The mixture was sonicated and cooled to 20 °C. Then, a toluene/anhydrous MeCN (1:1) solution of *cis*-[PtCl_2_(dmso)_2_] (12.0 mM, 500 µL) was added to the mixture with stirring. The mixture was stirred at 20 °C for 1 h. The solids were collected by centrifugation and rinsed with toluene/anhydrous MeCN (1:1, 1000 µL) twice by redispersion and centrifugation. The sediment was redispersed in toluene (1000 µL) and subjected to dispersion-state analyses. For powder-state analyses, the solids were collected by centrifugation and dried *in vacuo*.

#### Synthesis of (CTA)_2_[PtCl_4_]

CTAC (970 mg) was dispersed in H_2_O (10 mL) in a 100 mL flask. An aqueous K_2_PtCl_4_ solution (402 mg in 50 mL) was added dropwise to the dispersion with stirring. The mixture was stirred at RT for 1.5 h in the dark. The precipitate was collected by centrifugation and rinsed with H_2_O (50 mL) twice by redispersion and centrifugation. The sediment was dispersed in CHCl_3_ (20 mL) with gentle heating. The undissolved solids and residual aqueous phase were removed by centrifugation. *n*-Hexane (20 mL) was added to the solution. The precipitate was collected by centrifugation and dried *in vacuo* to give pale orange solids of (CTA)_2_[PtCl_4_] (806 mg, 92% yield).

SEM–EDX: Cl/Pt atomic ratio = 3.91(11) (with *φρZ* correction from the calculated C, H, and N composition)

#### Synthesis of PtSA@CdSe

A toluene dispersion of CdSe NPLs (37.9 mM in CdSe units, 79.2 µL), toluene (171 µL), and anhydrous MeOH (250 µL) were combined in a 2 mL microtube. The mixture was sonicated and heated to 30 °C. A toluene/anhydrous MeOH (1:1) solution of (CTA)_2_[PtCl_4_] (12.0 mM, 500 µL) was added to the mixture while stirring. The mixture was stirred at 30 °C for 1 h. The solids were collected by centrifugation and rinsed with toluene/anhydrous MeOH (1:1, 1000 µL) twice by redispersion and centrifugation. The sediment was redispersed in ^*n*^OctNH_2_/OAM (1:1, 1000 µL) by sonication. The dispersion was stirred at 30 °C for 1 h. The solids were collected by centrifugation and rinsed with ^*n*^OctNH_2_/OAM (1:1, 1000 µL) twice by redispersion and centrifugation. The sediment was redispersed in toluene (1000 µL) and transferred to a new 2 mL microtube. The solids were collected by centrifugation and rinsed with toluene (1000 µL) twice by redispersion and centrifugation. The sediment was redispersed in toluene (1000 µL) and subjected to dispersion-state analyses. For powder-state analyses, the solids were collected by centrifugation and dried *in vacuo*.

#### Synthesis of PtSA@CdSe/PtSA

A toluene dispersion of PtSA@CdSe (3 mM of Se, 1000 µL) was centrifuged. The sediment was redispersed in a toluene/anhydrous MeCN (1:1) solution of *cis*-[PtCl_2_(dmso)_2_] (12.0 mM, 1000 µL) by sonication. The dispersion was stirred at 20 °C for 1 h. The solids were collected by centrifugation and rinsed with toluene/anhydrous MeCN (1:1, 1000 µL) twice by redispersion and centrifugation. The sediment was redispersed in toluene (1000 µL) and subjected to dispersion-state analyses. For powder-state analyses, the solids were collected by centrifugation and dried *in vacuo*.

### Ligand exchange with MUA

#### Ligand exchange of PtSA/CdSe and PtSA@CdSe/PtSA with MUA

MUA (101 mg) and KOH (97.0 mg) were dissolved in anhydrous MeOH (1.80 mL). A toluene dispersion of PtSA/CdSe or PtSA@CdSe/PtSA (3 mM of Se, 1000 µL) was centrifuged. The sediment was redispersed in the MeOH solution of MUA and KOH (1000 µL) by sonication. The dispersion was stirred at 20 °C for 1 h. The solids were collected by centrifugation and rinsed with MeOH (1000 µL) twice by redispersion and centrifugation. The sediment was redispersed in MeOH (1000 µL) and subjected to dispersion-state analyses. For powder-state analyses, the solids were collected by centrifugation and dried *in vacuo*. For photocatalysis, the solids were collected by centrifugation and used without drying.

#### Ligand exchange of PtSA@CdSe with CdBr_2_ and MUA

MUA (232 mg) and KOH (236 mg) were dissolved in H_2_O (4.38 mL). A toluene dispersion of PtSA@CdSe (3 mM of Se, 1000 µL) was centrifuged. The sediment was rinsed with a toluene/anhydrous MeCN/DMSO (45:45:10) solution of CdBr_2_·4H_2_O (60 mM, 1000 µL) three times and with toluene/anhydrous MeCN (1:1, 1000 µL) twice by redispersion and centrifugation. The sediment was dispersed in the aqueous solution of MUA and KOH (1000 µL). The dispersion was stirred at 20 °C for 1 h. The solids were collected by centrifugation and rinsed with MeOH (1000 µL) twice by redispersion and centrifugation. The sediment was redispersed in H_2_O (1000 µL) and subjected to dispersion-state analyses and photocatalysis. For powder-state analyses, the solids were collected by centrifugation, rinsed with MeOH (1000 µL) twice, and dried *in vacuo*.

#### Ligand exchange of CdSe NPLs with CdBr_2_ and MUA

MUA (232 mg) and KOH (236 mg) were dissolved in H_2_O (4.38 mL). A toluene dispersion of CdSe NPLs (37.9 mM of Se, 79.2 µL) was combined with toluene (146 µL), anhydrous MeCN (225 µL), DMSO (50.0 µL), and toluene/anhydrous MeCN/DMSO (45:45:10) solution of CdBr_2_·4H_2_O (120 mM, 500 µL). The solids were collected by centrifugation and rinsed with toluene/anhydrous MeCN (1:1, 1000 µL) twice by redispersion and centrifugation. The sediment was dispersed in the aqueous solution of MUA and KOH (1000 µL). The dispersion was stirred at 20 °C for 1 h. The solids were collected by centrifugation and rinsed with MeOH (1000 µL) twice by redispersion and centrifugation. The sediment was redispersed in MeOH (1000 µL) and subjected to dispersion-state analyses. For powder-state analyses, the solids were collected by centrifugation and dried *in vacuo*. For photocatalysis, the solids were collected by centrifugation and used without drying.

#### Photocatalytic hydrogen evolution

Photocatalytic hydrogen evolution was quantified with a lab-fabricated online-flow sampling system comprising a glass reactor connected to a gas chromatographer (GC-2004, Shimadzu, with argon as the carrier gas) equipped with a thermal conductivity detector. The photocatalyst (2.7 µmol) was dispersed in 50 mL of an aqueous solution containing TEOA (10 mL) as a sacrificial reagent. The accurate catalyst concentration of each sample was measured by ICP–OES and showed a small variation within 41–51 µmol dm^−3^. These values were used to normalise the photocatalytic activity. The reaction mixture was vigorously stirred at a high rate throughout the process to prevent the agglomeration of catalyst particles. Before light irradiation, the dispersion was bubbled with argon for 1 h, then the system was sealed and maintained under an argon flow for 10 min to remove oxygen. Irradiation was conducted with a 300 W Xe lamp (Cermax PE300BF, Excelitas Technology) equipped with a long-pass filter (λ > 422 nm). The irradiance at the reactor was adjusted to be 1000 W/m^2^. The reaction temperature was maintained at 30 °C with a cooling plate and block. The flow rate of argon was ~3.9 mL/min.

## Supplementary information

Supplementary Information

## Data Availability

All data are available from the authors upon request. Source data are provided with this paper.

## Source data

Source Data
