# Supplementary material for: Location-selective immobilisation of single-atom catalysts on the surface or within the interior of ionic nanocrystals using coordination chemistry
Source: Nat Commun. 2023 Jul 15;14:4241. doi: 10.1038/s41467-023-40003-8 (PMC10349889; doi:10.1038/s41467-023-40003-8)
Supplement: Supplementary file 1 — Supplementary Information [file 41467_2023_40003_MOESM1_ESM.pdf]

*Supplementary Information for*

# Location-selective immobilization of single-atom catalysts on the surface or within the interior of ionic nanocrystals using coordination chemistry

Kenichi Endo, Masaki Saruyama and Toshiharu Teranishi*

*Corresponding author. E-mail: teranisi@scl.kyoto-u.ac.jp (T.T.)

Supplementary Figures and Tables ........ 2
CdSe NPLs ........ 2
PtSA/CdSe ........ 4
Reaction with other Pt precursors ........ 9
PtSA@CdSe ........ 11
PtSA@CdSe/PtSA ........ 15
Vis–DR spectra comparison ........ 17
Ligand exchange with MUA ........ 18
Photocatalysis ........ 23
XAFS Analyses ........ 30
Determination of the amplitude reduction factor for FT-EXAFS curve-fitting ........ 30
FT-EXAFS of PtSA/CdSe ........ 31
FT-EXAFS of PtSA@CdSe ........ 33
XANES ........ 35
Supplementary References ........ 36

# Supplementary Figures and Tables

## CdSe NPLs

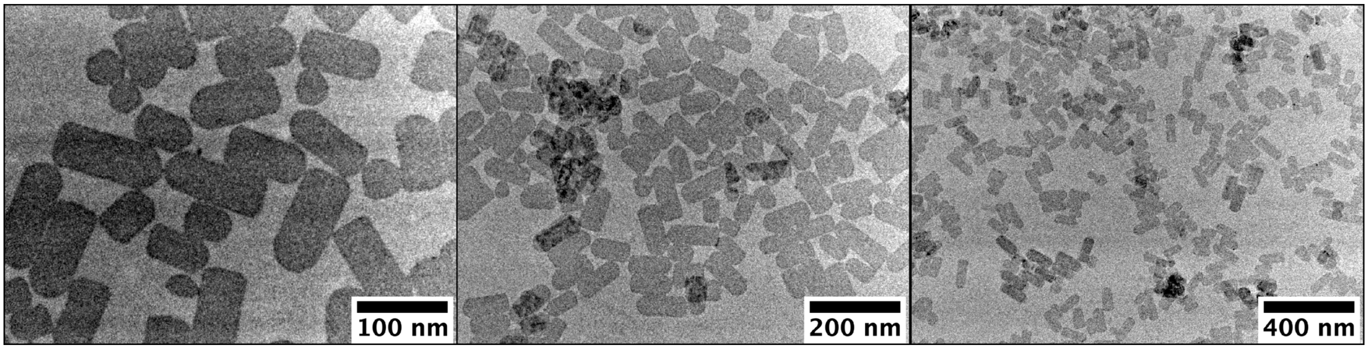


**Figure S1.** TEM images of CdSe NPLs.

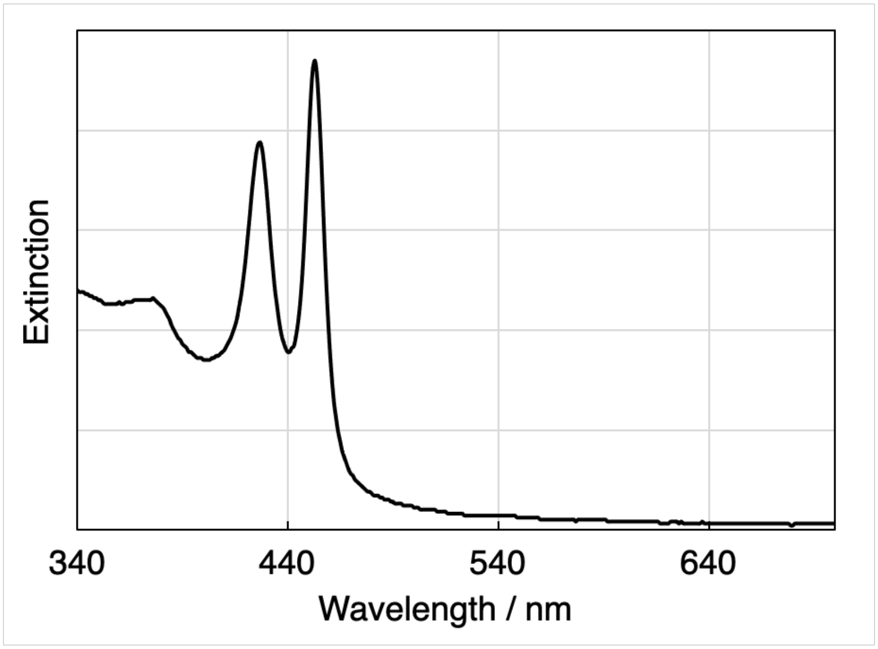


**Figure S2.** UV–Vis extinction spectrum of CdSe NPLs in $CHCl_3$/OAM.

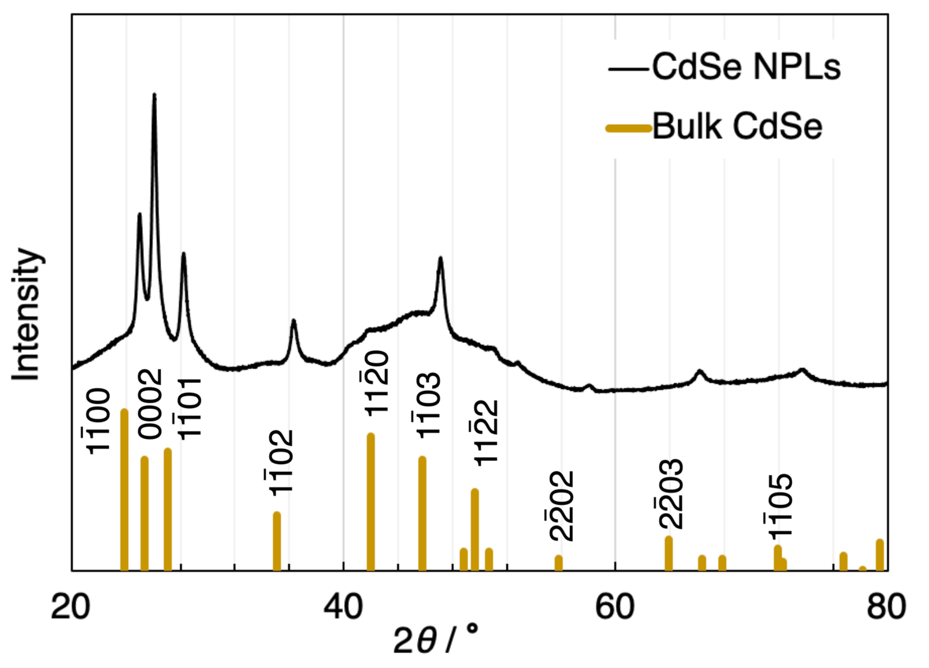


**Figure S3.** XRD patterns of CdSe NPLs and bulk wurtzite-type CdSe (ICDD No. 00-008-0459) measured with CuKα irradiation.

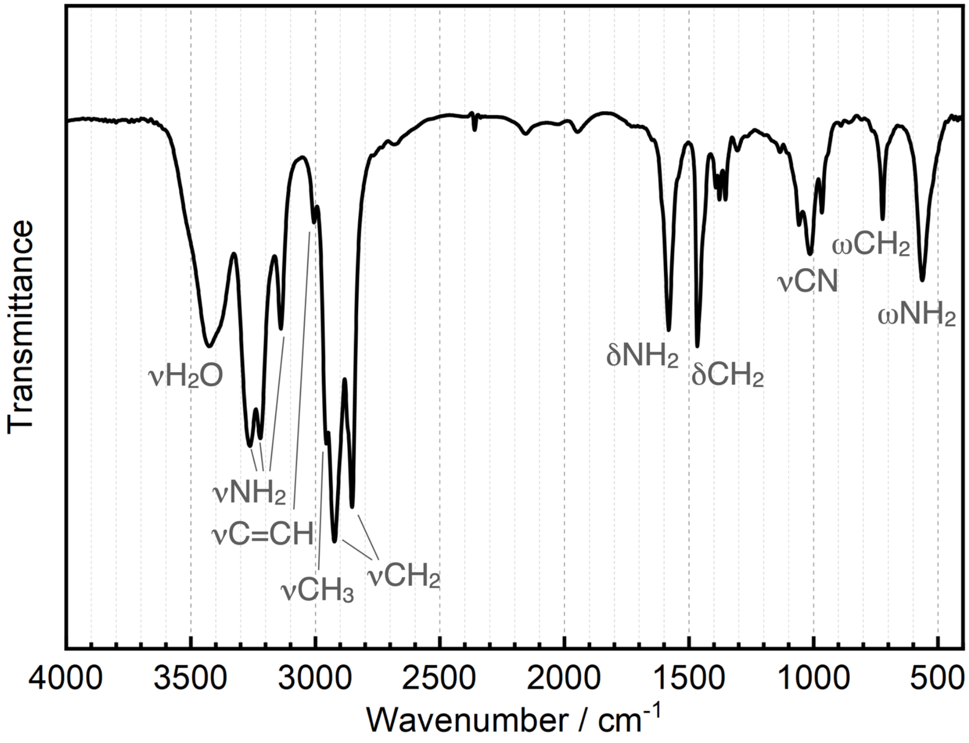


**Figure S4.** IR spectrum of CdSe NPLs. Symbols: ν, stretch; δ, scissors; ω, wagging.

## PtSA/CdSe

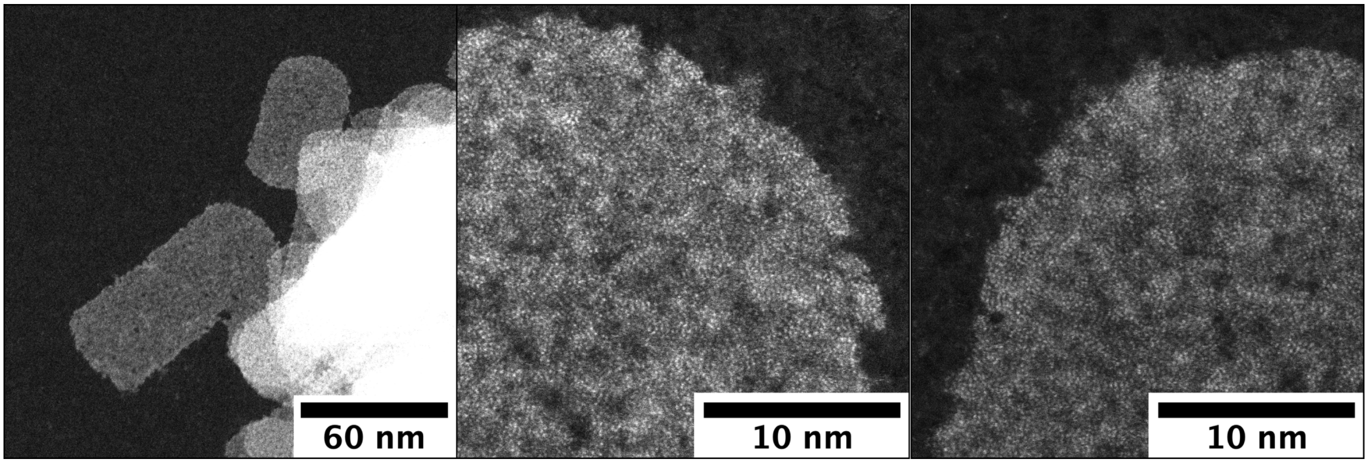


**Figure S5.** HAADF-STEM images of PtSA/CdSe. Samples were severely susceptible to electron beam damage. Fragmentation, hole formation, and Cd nanoparticle formation were observed during measurement. The ordered atomic patterns of CdSe were observed only for some regions.

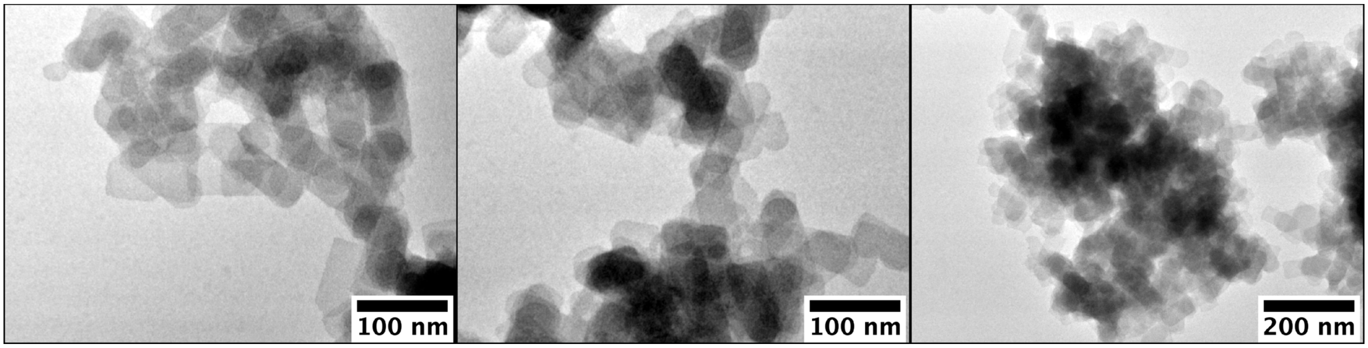


**Figure S6.** TEM images of PtSA/CdSe.

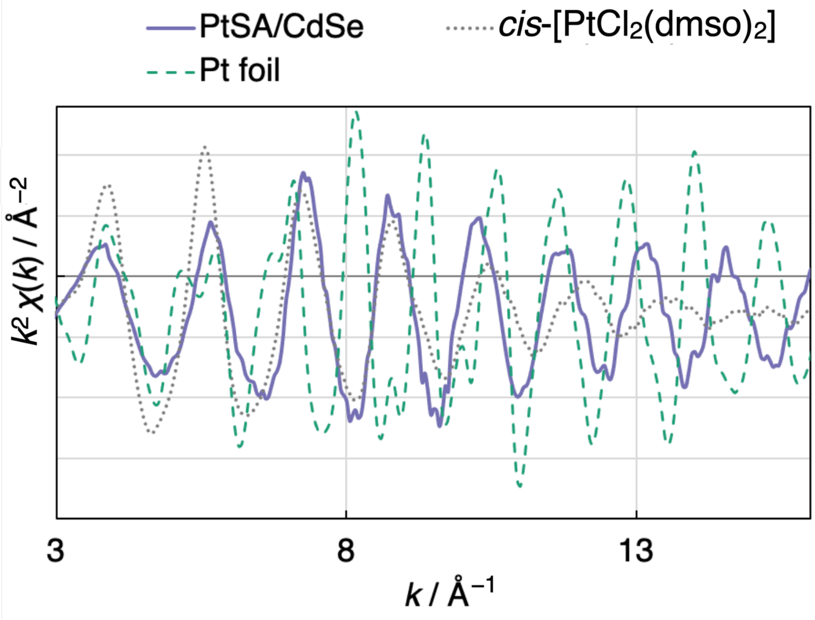


**Figure S7.** $k^2$-weighted EXAFS oscillations of PtSA/CdSe, *cis*-[$PtCl_2(dmso)_2$], and Pt foil.

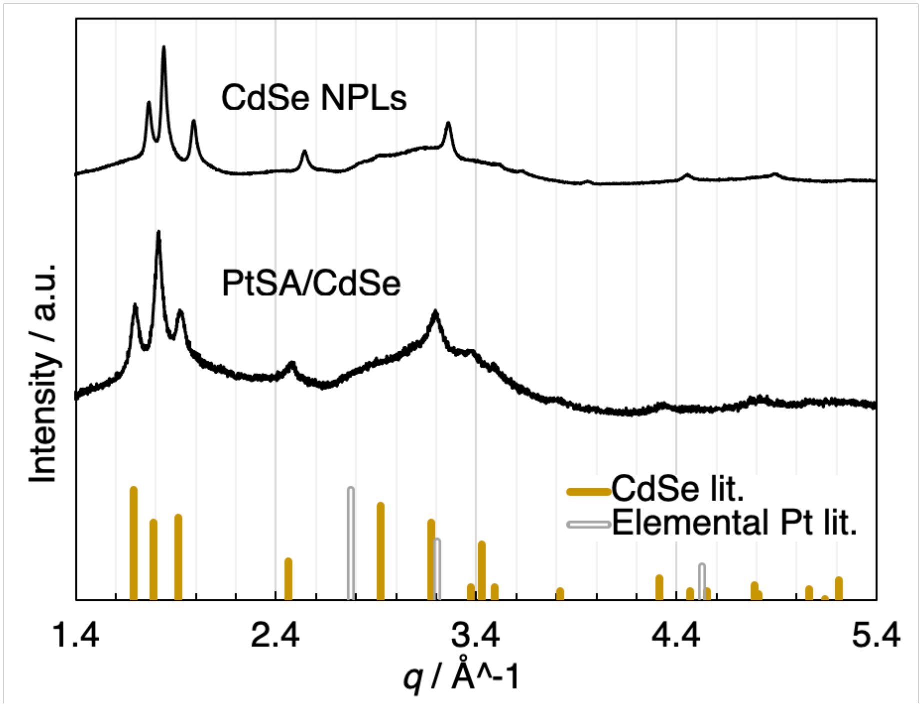


**Figure S8.** XRD patterns of CdSe NPLs, PtSA/CdSe, and references from literature (ICDD No. 00-008-0459 for wurtzite-type CdSe and No. 00-004-0802 for elemental Pt). CdSe NPLs were measured using CuKα radiation, while PtSA/CdSe was measured using synchrotron radiation because it decomposes under CuKα radiation. The XRD pattern for PtSA/CdSe obtained with synchrotron radiation is essentially the same as that obtained with CuKα radiation except for the absence of decomposition products.

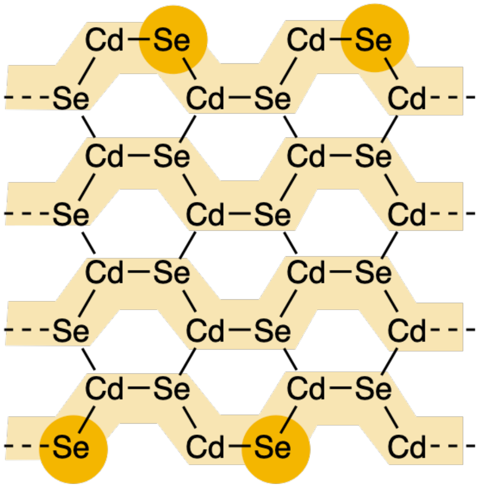


**Figure S9.** Schematic representation of CdSe NPLs. Corrugated layer structure and exposed Se atoms are highlighted.

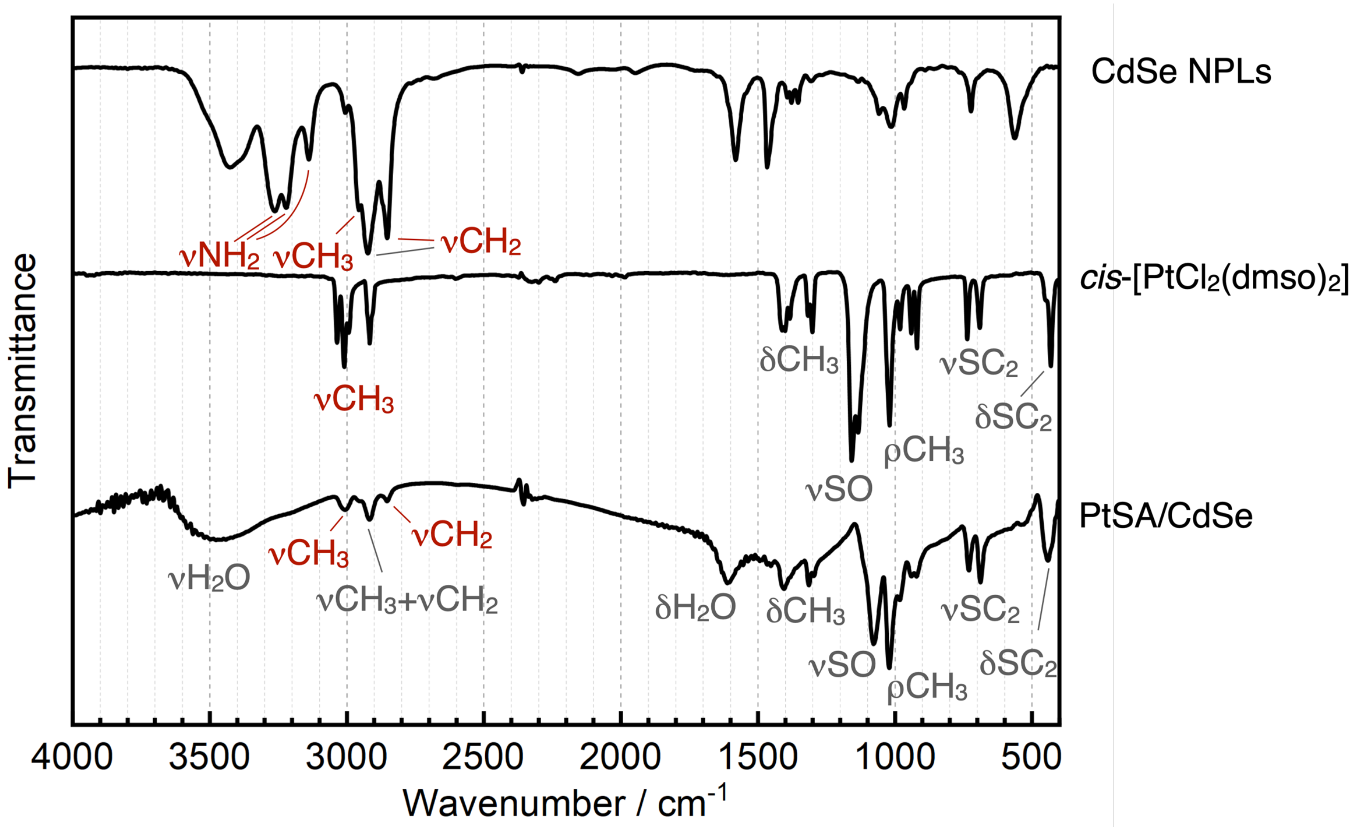


**Figure S10.** IR spectra of PtSA/CdSe along with *cis*-$[PtCl_2(dmso)_2]$ and CdSe NPLs. Symbols: ν, stretch; δ, scissors; ρ, rocking. The vibrational assignment of *cis*-$[PtCl_2(dmso)_2]$ is based on the literature.[1]

**Table S1.** S–O stretching wavenumbers of related compounds in IR spectroscopy.

| Compound | $\tilde{\nu}$ / cm$^{-1}$ | Cause of shift | Ref. |
|---|---|---|---|
| **PtSA/CdSe** | 1076 | Coordinated to Pt via S and, coordinated to Cd or hydrogen-bonded via O | This work |
| ***cis*-[PtCl$_2$(dmso)$_2$]** | 1157, 1134 | Coordinated with Pt via S | This work |
| ***cis*-[PtCl$_2$(dmso)$_2$]** | 1151, 1128 | Coordinated with Pt via S | [1] |
| **DMSO (liquid)** | 1070 | Pure state | [1] |
| **DMSO (aq)** | 1024 | Hydrogen-bonded via O | [1] |
| **CdCl$_2$·DMSO** | 950 | Coordinated with Cd via O | [2] |

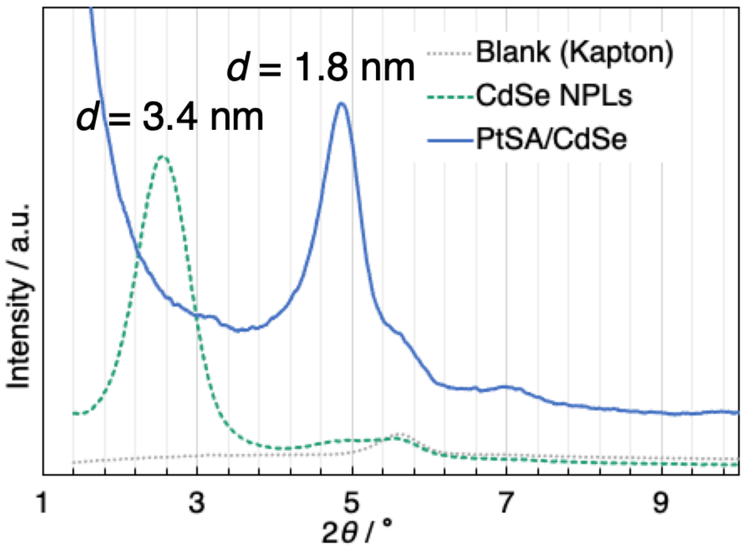


**Figure S11.** SAXS patterns of CdSe NPLs, PtSA/CdSe, and blank (scattering from a Kapton bag). *d*-spacings calculated at peak maxima are shown.

**Table S2.** 2D lattice constants (Å) of CdSe NPLs and PtSA/CdSe calculated from XRD data.

| Entry | $[1\bar{1}00]$ | [0001] |
|---|---|---|
| **CdSe NPLs** | 3.56 | 6.84 |
| **PtSA/CdSe** | 3.70 | 6.92 |

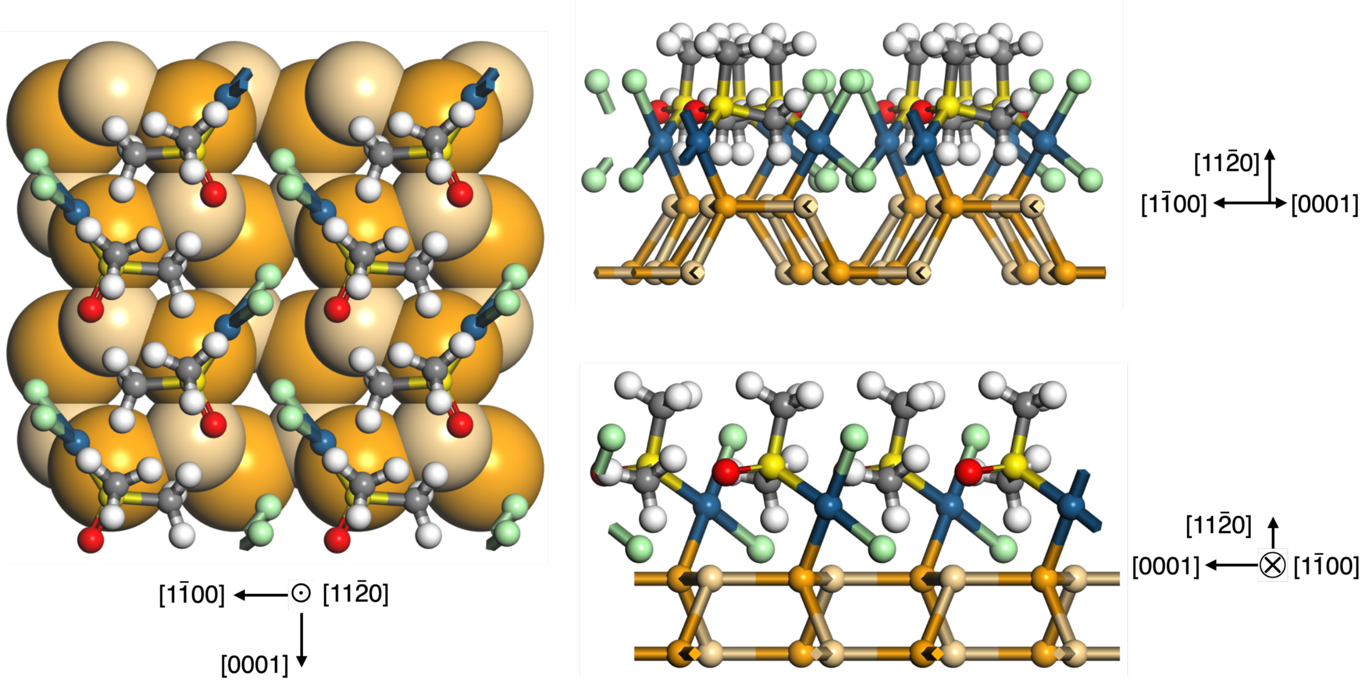


**Figure S12.** Surface structure model of PtSA/CdSe based on its XRD data and the reported crystal structure of *cis*-$[PtCl_2(dmso)_2]$ viewed from three directions. Each view is drawn for two unit cells in both surface directions with the top monolayer of a CdSe NPL. The CdSe section in the $(11\bar{2}0)$ view is shown in a space-filling style, and the others are shown in a ball-and-stick style.

## Reaction with other Pt precursors

**Table S3.** ICP-OES and SEM-EDX results of CdSe NPLs, PtSA/CdSe, and PtSA adsorption attempts with other Pt sources. Cd/Se and Pt/Se atomic ratios were determined by ICP-OES. Cl/Se and S/Se atomic ratios were determined by a combination of ICP-OES (Cd/Se) and SEM-EDX (Cl/Cd, S/Cd). N.D.: not detected.

| Entry | Cd/Se | Pt/Se | Cl/Se | S/Se |
|---|---|---|---|---|
| **CdSe NPLs** | 1.002(10) | N.D. | N.D. | N.D. |
| **PtSA/CdSe** | 0.998(10) | 0.251(3) | 0.537(7) | 0.242(9) |
| **with $(CTA)_2[PtCl_4]$ (in DCM)** | 0.98(7) | 0.065(3) | 0.183(15) | N.D. |
| **with *cis*-$[PtCl_2(NCPh)_2]$** | 0.999(10) | 0.01305(13) | 0.0507(10) | N.D. |
| **with $H_2PtCl_6$** | 0.872(2) | 0.467(5) | 1.814(18) | N.D. |

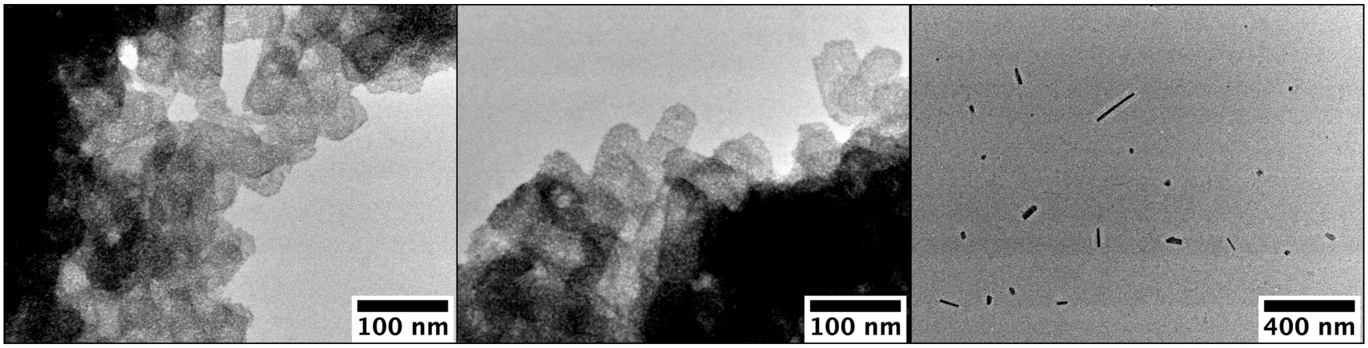


**Figure S13.** TEM images of the product obtained by the reaction of CdSe NPLs with $H_2PtCl_6$.

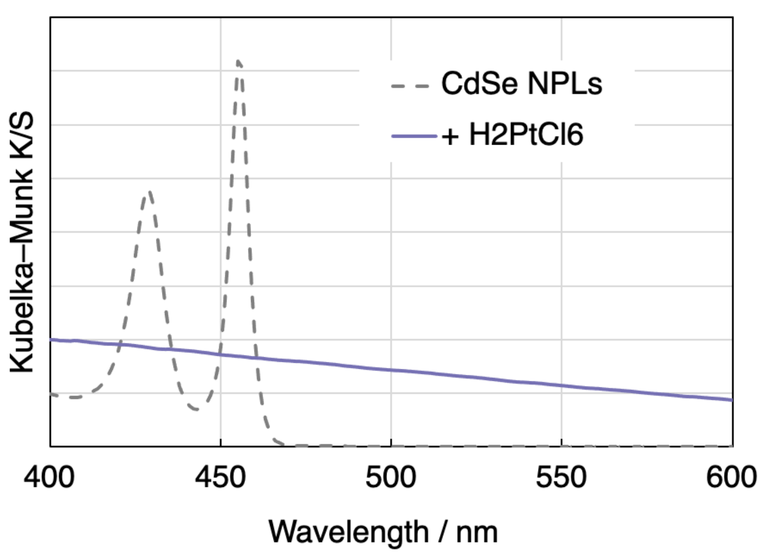


**Figure S14.** Vis-DR spectra of the product obtained by the reaction of CdSe NPLs with $H_2PtCl_6$ and the original CdSe NPLs (not normalized, dispersion with the same concentration).

## PtSA@CdSe

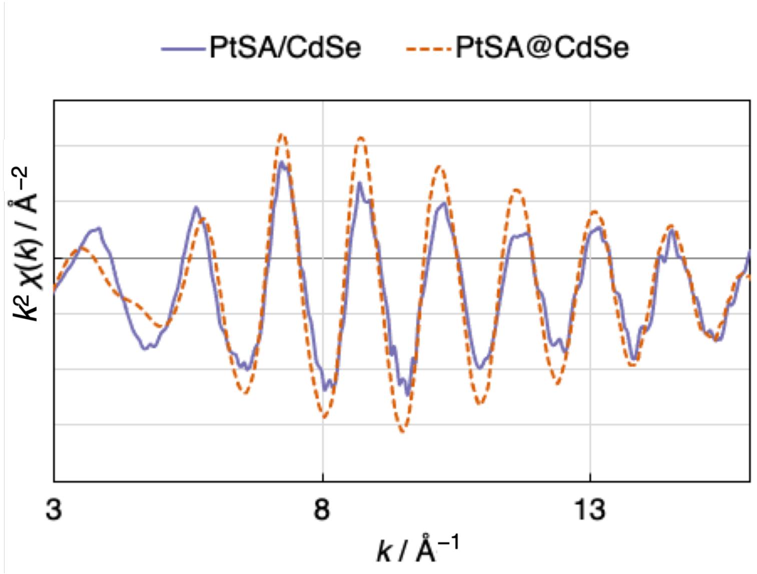


**Figure S15.** $k^2$-weighted EXAFS oscillations of PtSA/CdSe and PtSA@CdSe.

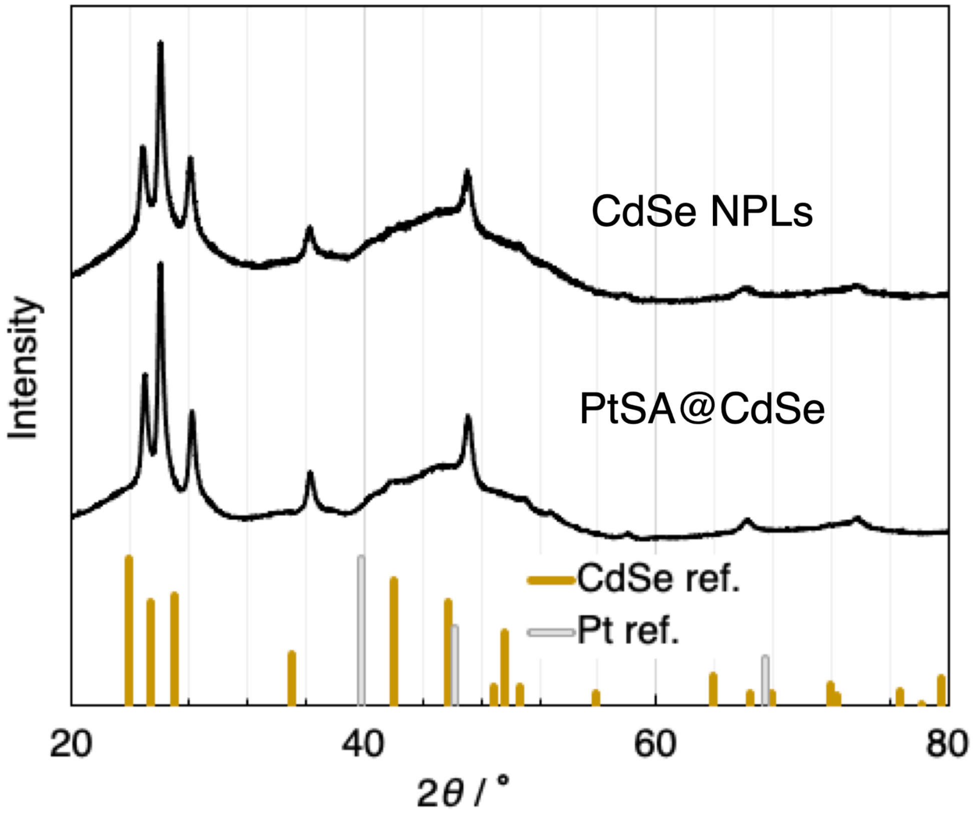


**Figure S16.** XRD patterns of CdSe NPLs, PtSA@CdSe, and references from literature (ICDD No. 00-008-0459 for wurtzite-type CdSe and No. 00-004-0802 for elemental Pt) measured with CuKα irradiation.

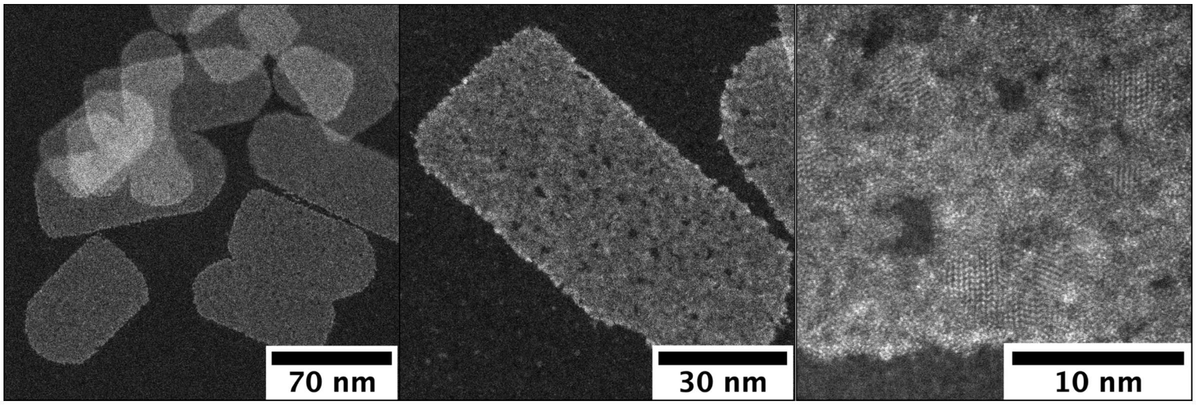


**Figure S17.** HAADF-STEM images of PtSA@CdSe. Samples were severely susceptible to electron beam damage. Fragmentation, hole formation, and Cd nanoparticle formation were observed during measurement. Ordered atomic patterns for CdSe were observed only for some regions.

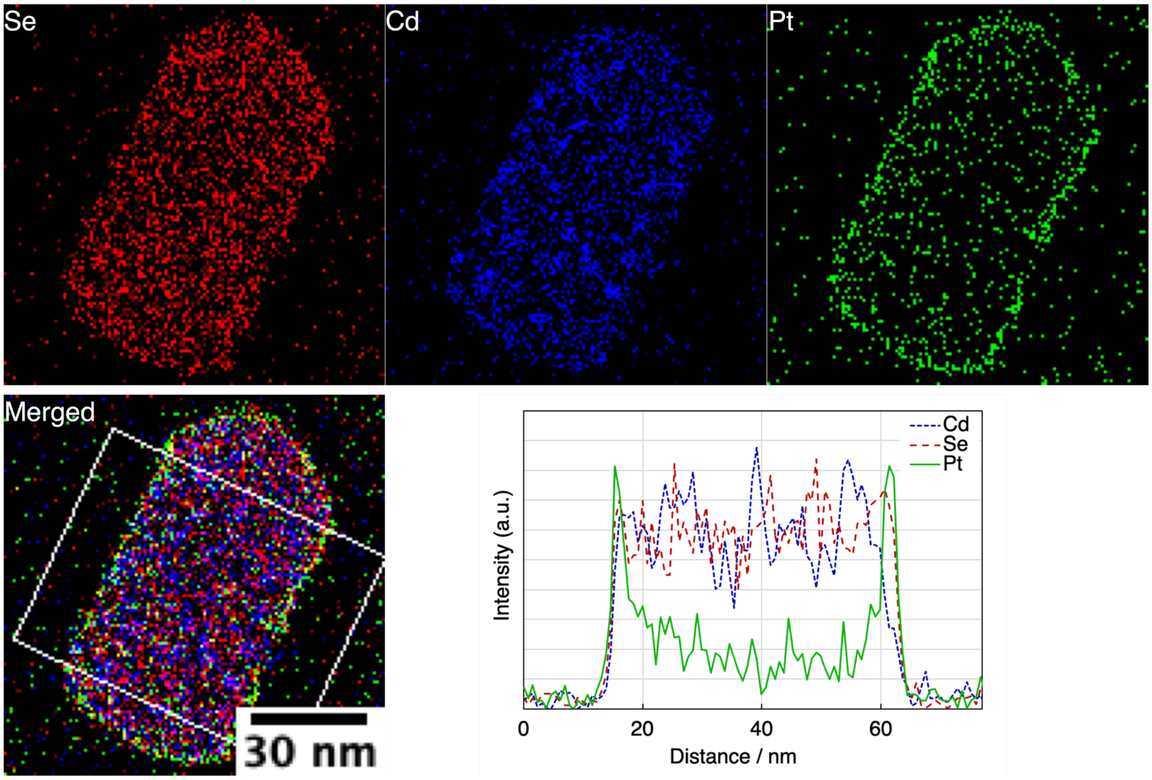


**Figure S18.** STEM–EDX maps of PtSA@CdSe. The graph shows the integrated signal intensity profiles along the short axis of an NPL. The white rectangle in the merged image shows the integration area. The samples were severely susceptible to electron beam damage, and Cd nanoparticles formed gradually during repeated scans.

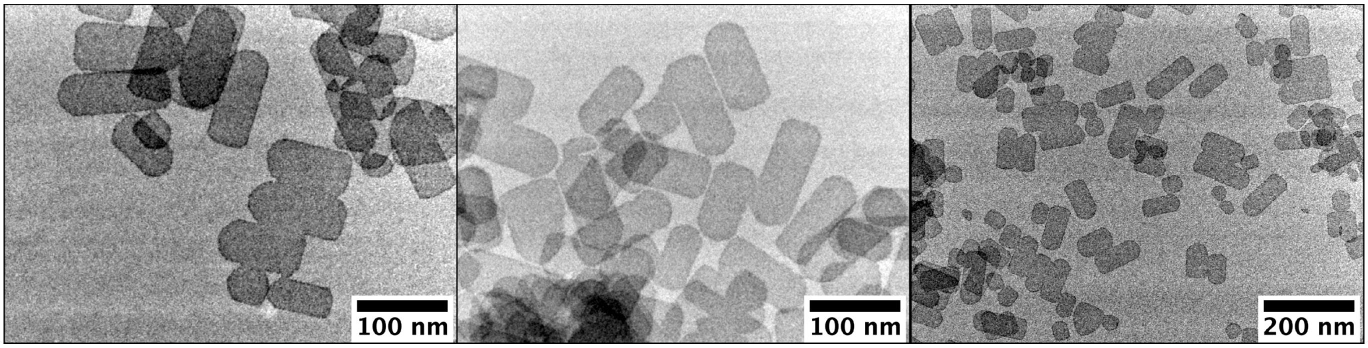


**Figure S19.** TEM images of PtSA@CdSe.

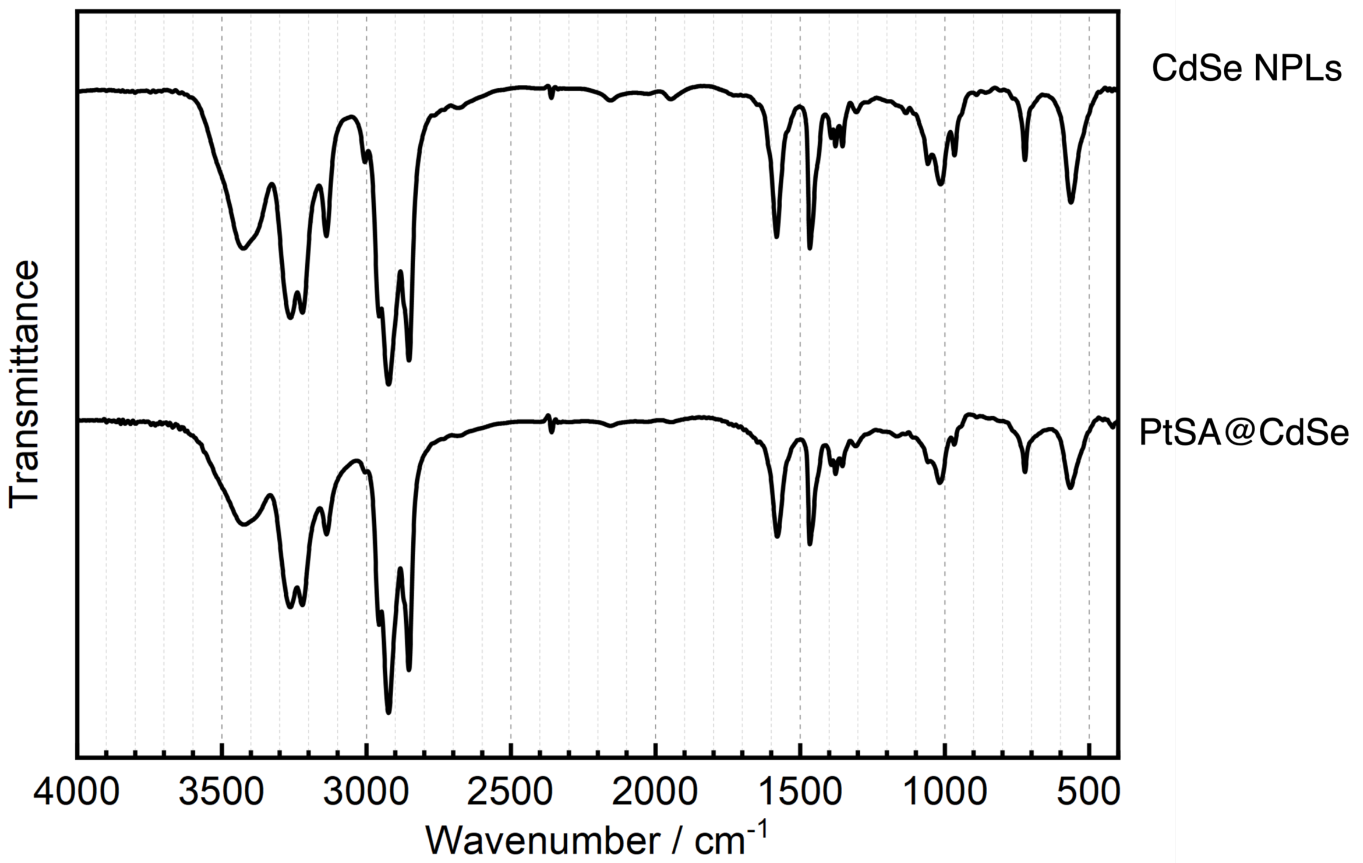


**Figure S20.** IR spectra of CdSe NPLs and PtSA@CdSe.

**Table S4.** Attempts to increase Pt loading in PtSA@CdSe and their product atomic ratios determined by ICP-OES and SEM-EDX.

| Sample name | $(CTA)_2[PtCl_4]$ amount (equiv.) | Reaction time (h) | Cd/Se | Pt/Se | Cl/Se | Pt valence |
|---|---|---|---|---|---|---|
| PtSA@CdSe-1 | 1 | 1 | 0.974(10) | 0.175(2) | 0.292(3) | 1.95(2) |
| PtSA@CdSe-2 | 2 | 1 | 0.88(9) | 0.333(12) | 0.43(5) | 2.0(3) |
| PtSA@CdSe-3 | 1 | 3 | 0.62(4) | 0.57(2) | 0.42(3) | 2.08(12) |

PtSA@CdSe-1 is prepared in a similar way to PtSA@CdSe but without amine treatment, which corresponds to entry 4 in Extended Data Table 1.

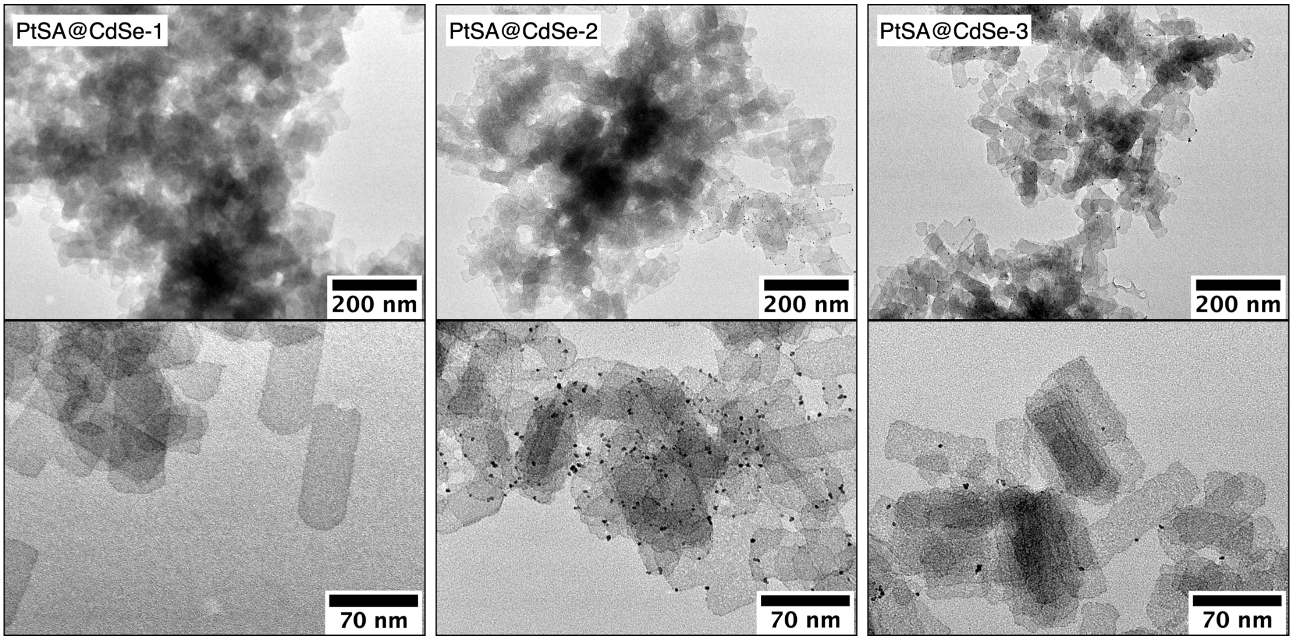


**Figure S21.** TEM images of attempts to increase Pt loading in PtSA@CdSe.

## PtSA@CdSe/PtSA

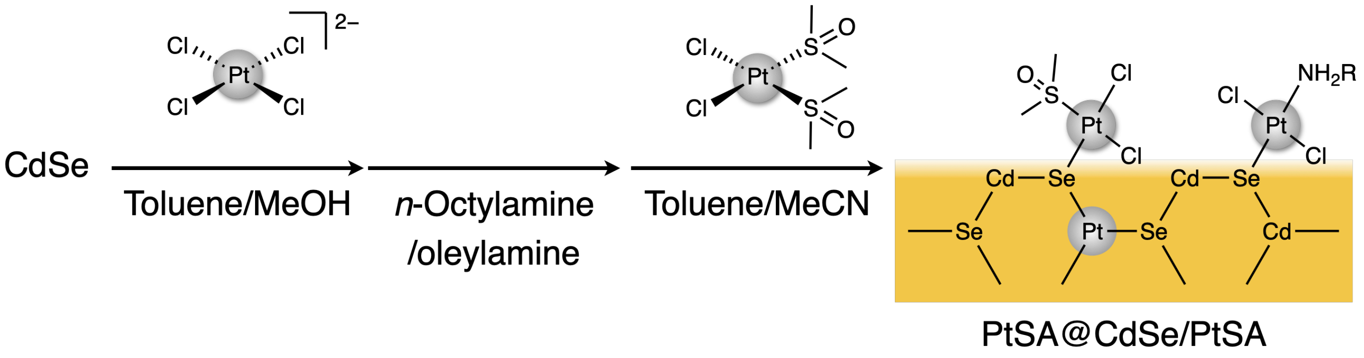


**Figure S22.** Stepwise reaction scheme for the hybrid adsorption–substitution loading of PtSAs and a schematic representation of the product PtSA@CdSe/PtSA.

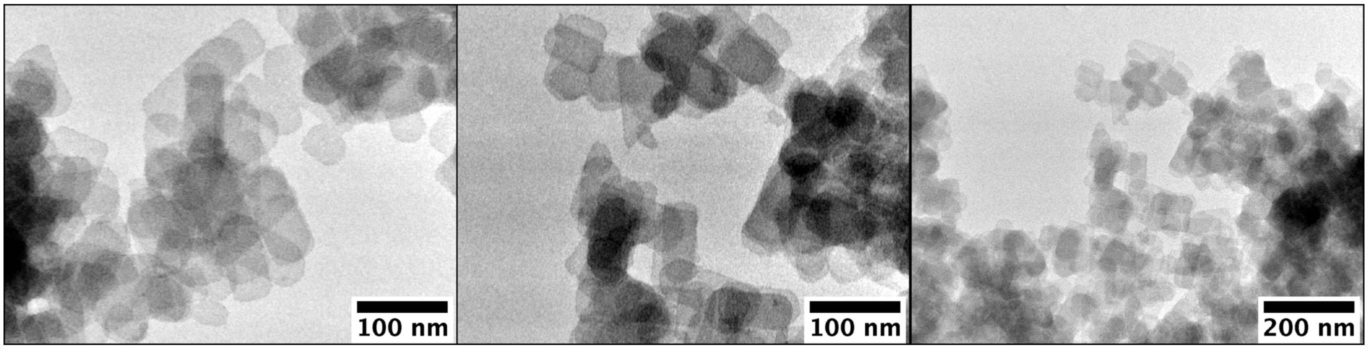


**Figure S23.** TEM images of PtSA@CdSe/PtSA.

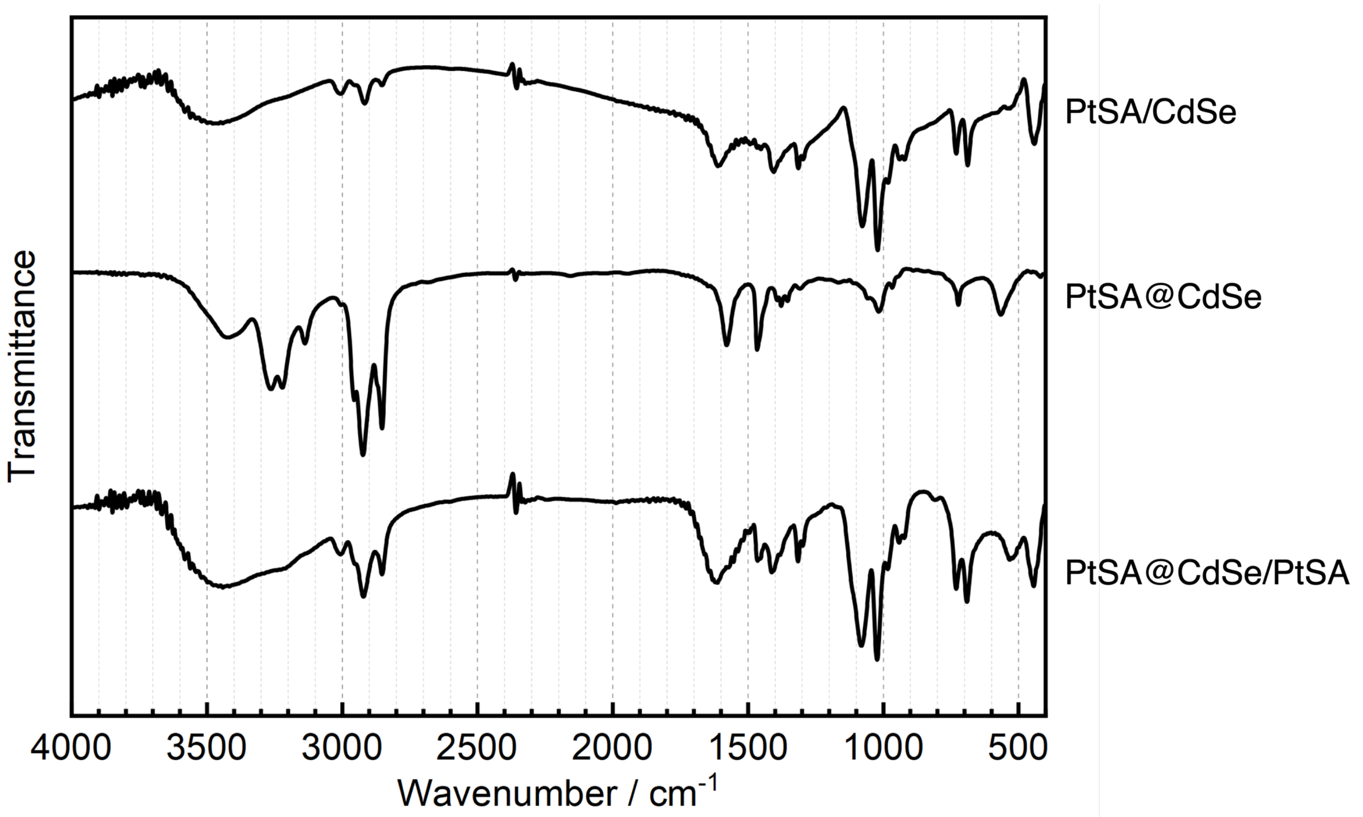


**Figure S24.** IR spectra of PtSA/CdSe, PtSA@CdSe, and PtSA@CdSe/PtSA.

## Vis–DR spectra comparison

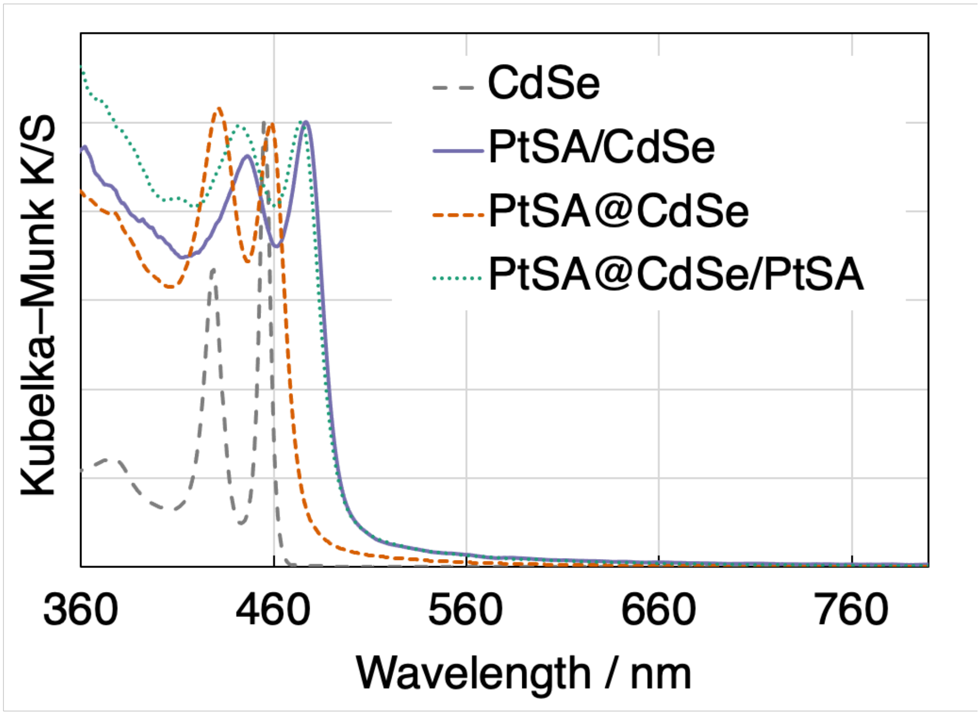


**Figure S25.** Vis–DR spectra of the original CdSe NPLs and the products, normalized at the first exciton peak maxima.

## Ligand exchange with MUA

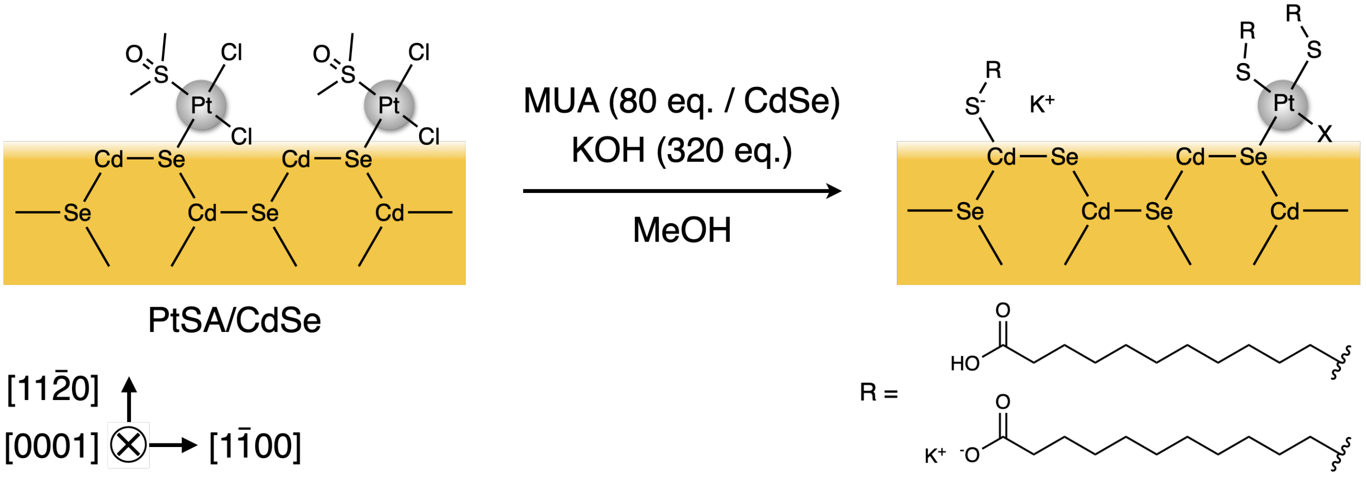


**Figure S26.** Reaction scheme showing ligand exchange of PtSA/CdSe and PtSA@CdSe/PtSA with MUA.

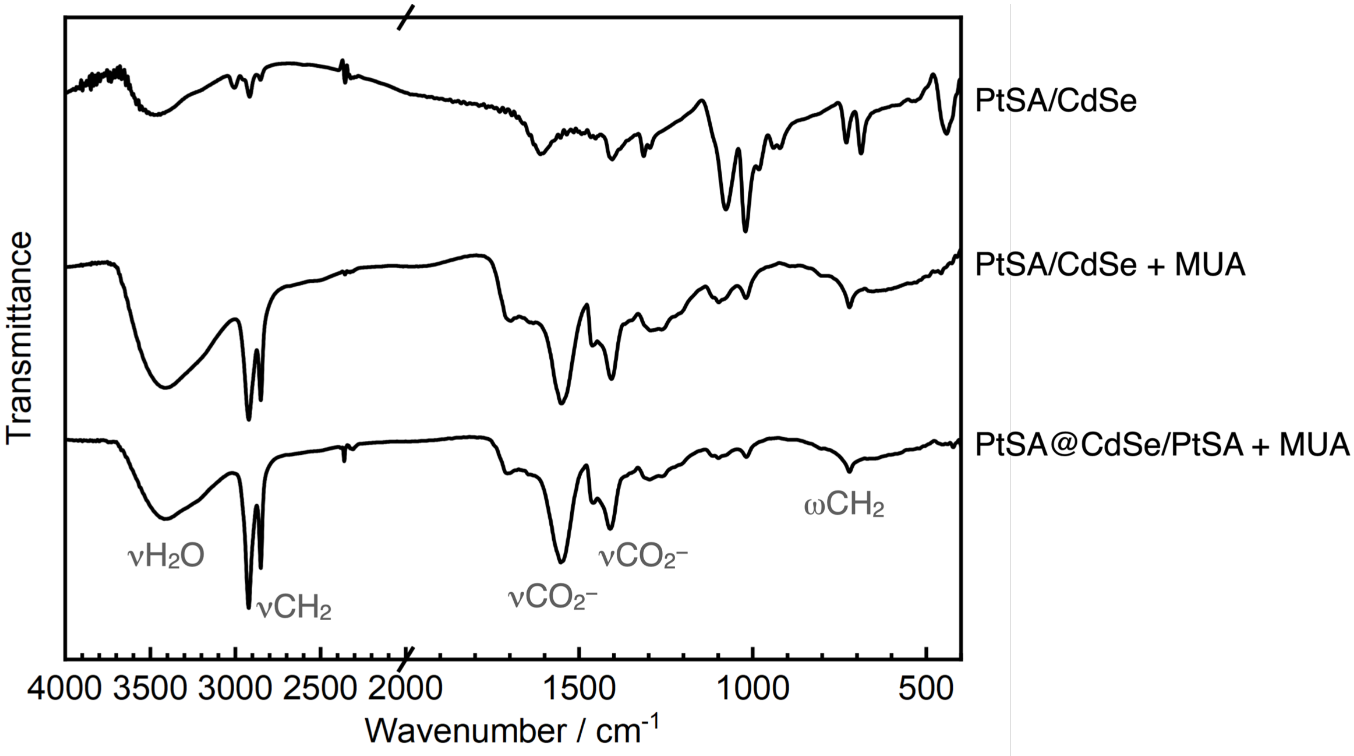


**Figure S27.** IR spectrum of PtSA/CdSe and spectra of PtSA/CdSe and PtSA@CdSe/PtSA after ligand exchange with MUA. Symbols: ν, stretch; ω, wagging.

**Table S5.** ICP–OES and SEM–EDX results of PtSA/CdSe and PtSA@CdSe/PtSA before and after ligand exchange with MUA. Cd/Se and Pt/Se atomic ratios were determined by ICP-OES. Cl/Se, S/Se and K/Se atomic ratios were determined by a combination of ICP-OES (Cd/Se) and SEM-EDX (Cl/Cd, S/Cd, K/Cd). N.D.: not detected.

| Entry | Cd/Se | Pt/Se | Cl/Se | S/Se | K/Se |
|---|---|---|---|---|---|
| **PtSA/CdSe** | 0.998(10) | 0.251(3) | 0.537(7) | 0.242(9) | 0.242(9) |
| **PtSA/CdSe + MUA** | 1.010(10) | 0.175(2) | 0.0143(8) | 0.492(14) | 0.165(3) |
| **PtSA@CdSe/PtSA** | 0.951(10) | 0.303(3) | 0.522(10) | 0.205(11) | N.D. |
| **PtSA@CdSe/PtSA + MUA** | 0.945(9) | 0.250(2) | | | |

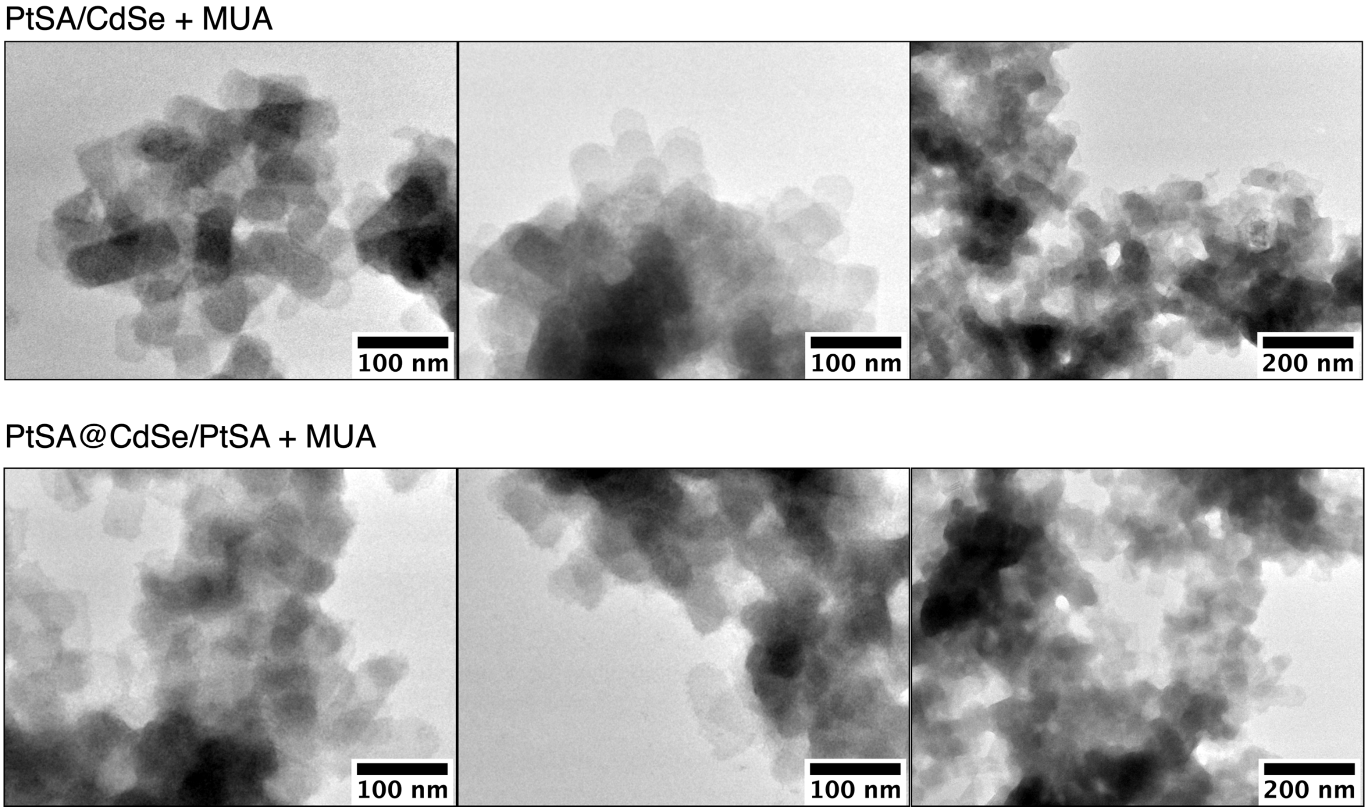


**Figure S28.** TEM images of PtSA/CdSe and PtSA@CdSe/PtSA after ligand exchange with MUA.

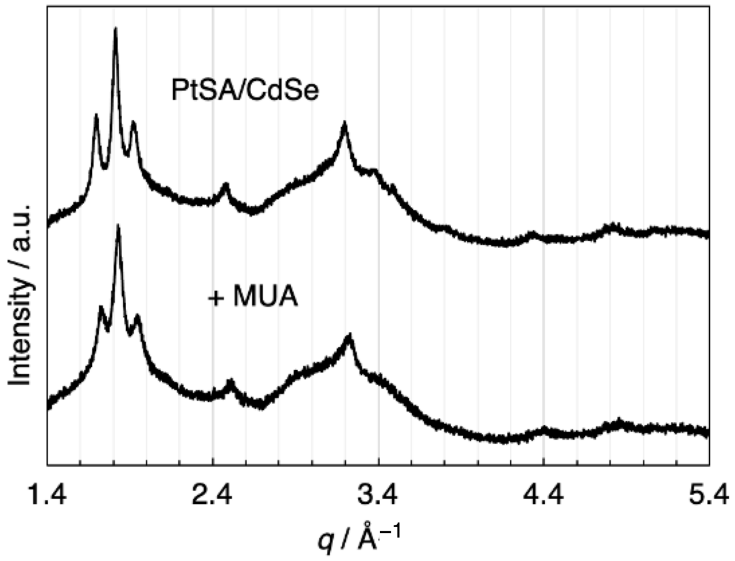


**Figure S29.** XRD patterns of PtSA/CdSe before and after ligand exchange with MUA.

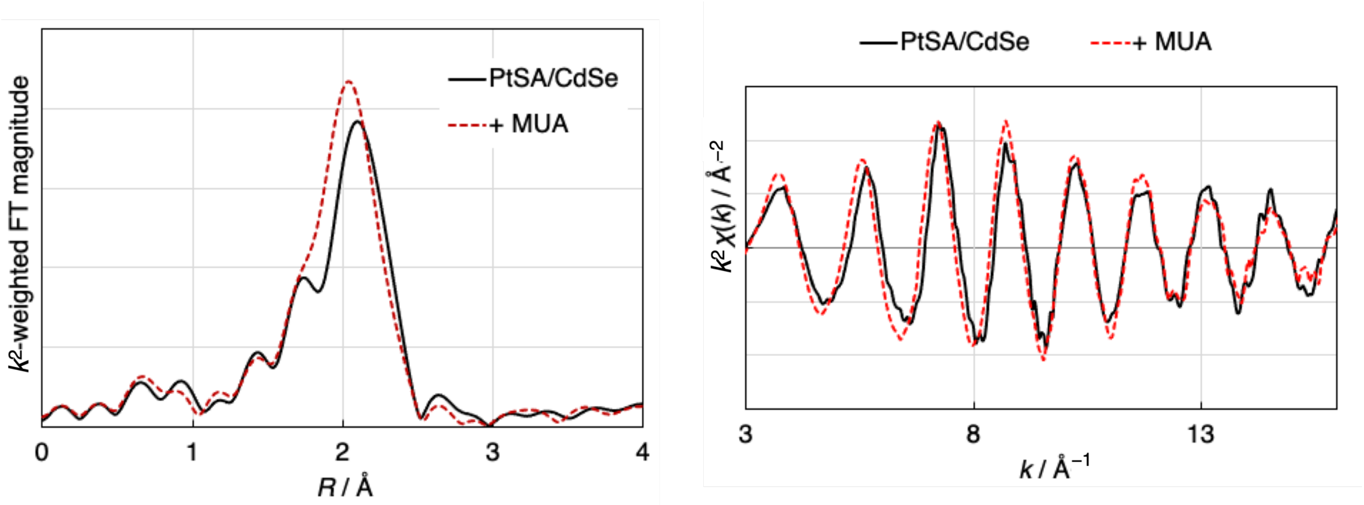


**Figure S30.** $k^2$-weighted EXAFS FT magnitude and oscillation of PtSA/CdSe before and after ligand exchange with MUA.

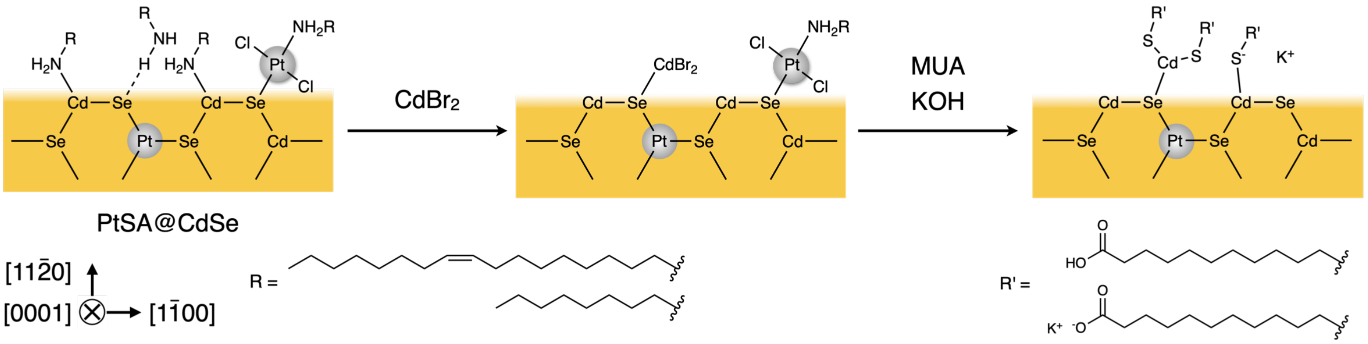


**Figure S31.** Reaction scheme of ligand exchange with $CdBr_2$ and MUA on CdSe NPLs and PtSA@CdSe.

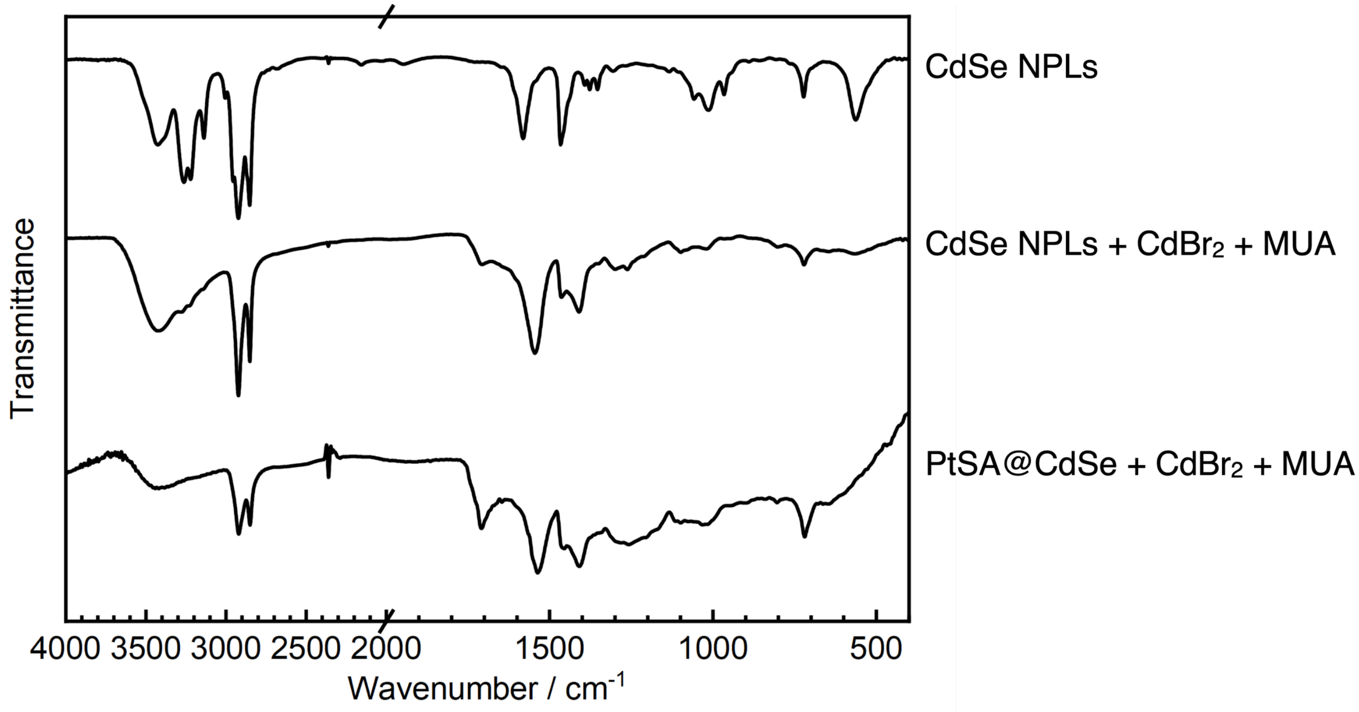


**Figure S32.** IR spectrum of pristine CdSe NPLs and spectra of CdSe NPLs and PtSA@CdSe after ligand exchange with $CdBr_2$ and MUA.

**Table S6.** ICP-OES and SEM-EDX results of CdSe NPLs and PtSA@CdSe before and after ligand exchange with $CdBr_2$ and MUA. Cd/Se and Pt/Se atomic ratios were determined by ICP-OES. Cl/Se, S/Se, K/Se and Br/Se atomic ratios were determined by a combination of ICP-OES (Cd/Se) and SEM-EDX (Cl/Cd, S/Cd, K/Cd, Br/Cd). N.D.: not detected.

| Entry | Cd/Se | Pt/Se | Cl/Se | S/Se | K/Se | Br/Se |
|---|---|---|---|---|---|---|
| **CdSe NPLs** | 1.002(10) | N.D. | N.D. | N.D. | N.D. | N.D. |
| **CdSe NPLs + $CdBr_2$ + MUA** | 1.208(12) | N.D. | N.D. | 0.312(7) | 0.048(4) | N.D. |
| **PtSA@CdSe** | 0.929(7) | 0.175(2) | 0.292(3) | N.D. | N.D. | N.D. |
| **PtSA@CdSe + $CdBr_2$ + MUA** | 1.203(12) | 0.0895(9) | | | | |

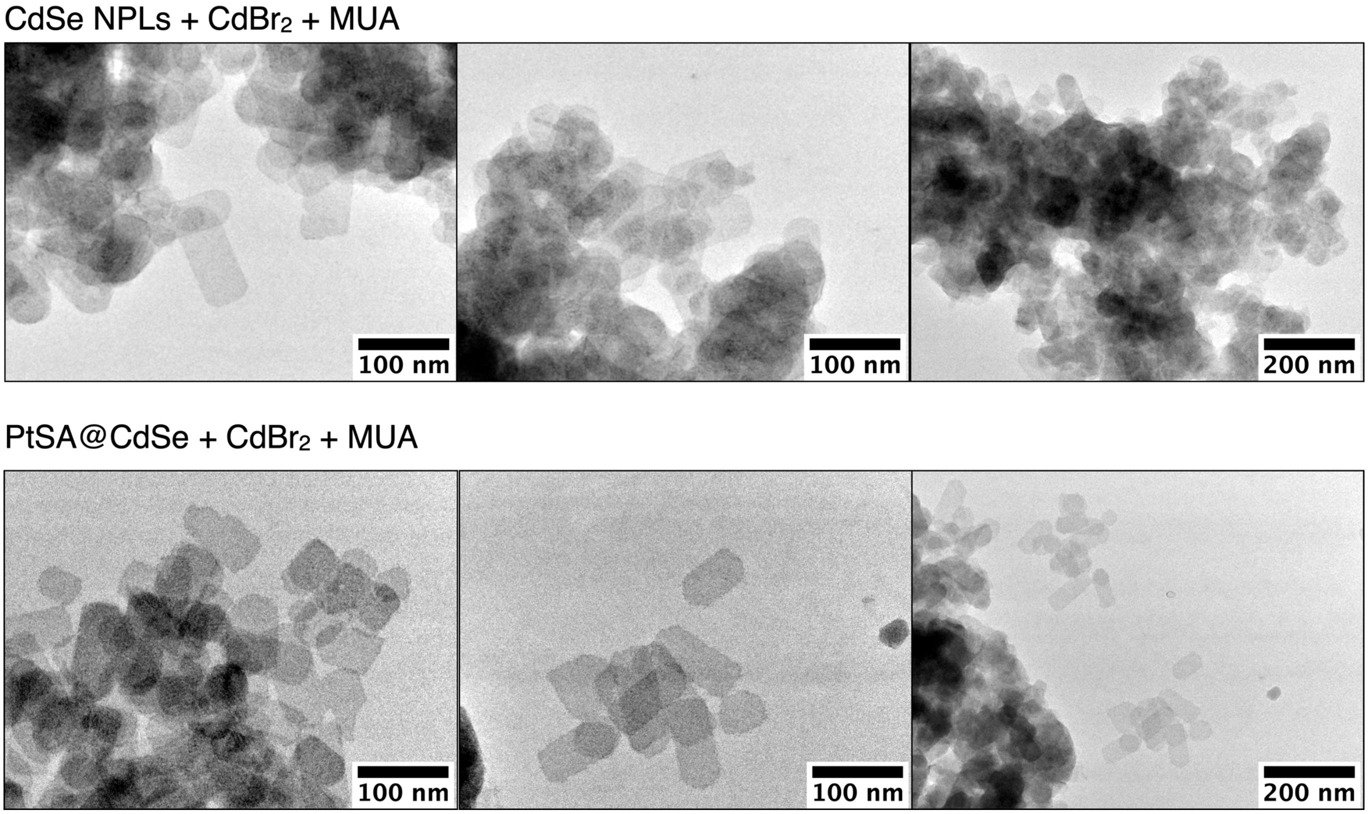


**Figure S33.** TEM images of CdSe NPLs and PtSA@CdSe after ligand exchange with $CdBr_2$ and MUA.

## Photocatalysis

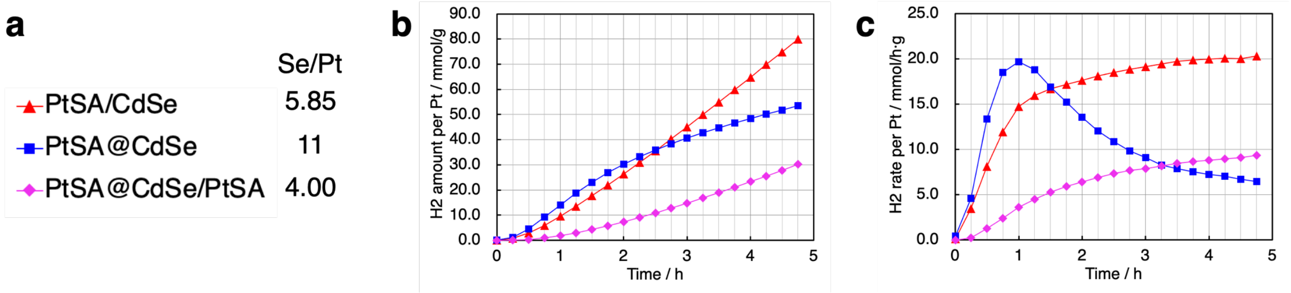


**Figure S34.** Photocatalytic hydrogen evolution normalized by Pt amount. (a) Legend and Se/Pt atomic ratios determined by ICP-OES. (b) Total evolved hydrogen amount over time. (c) Change in hydrogen evolution rate over time.

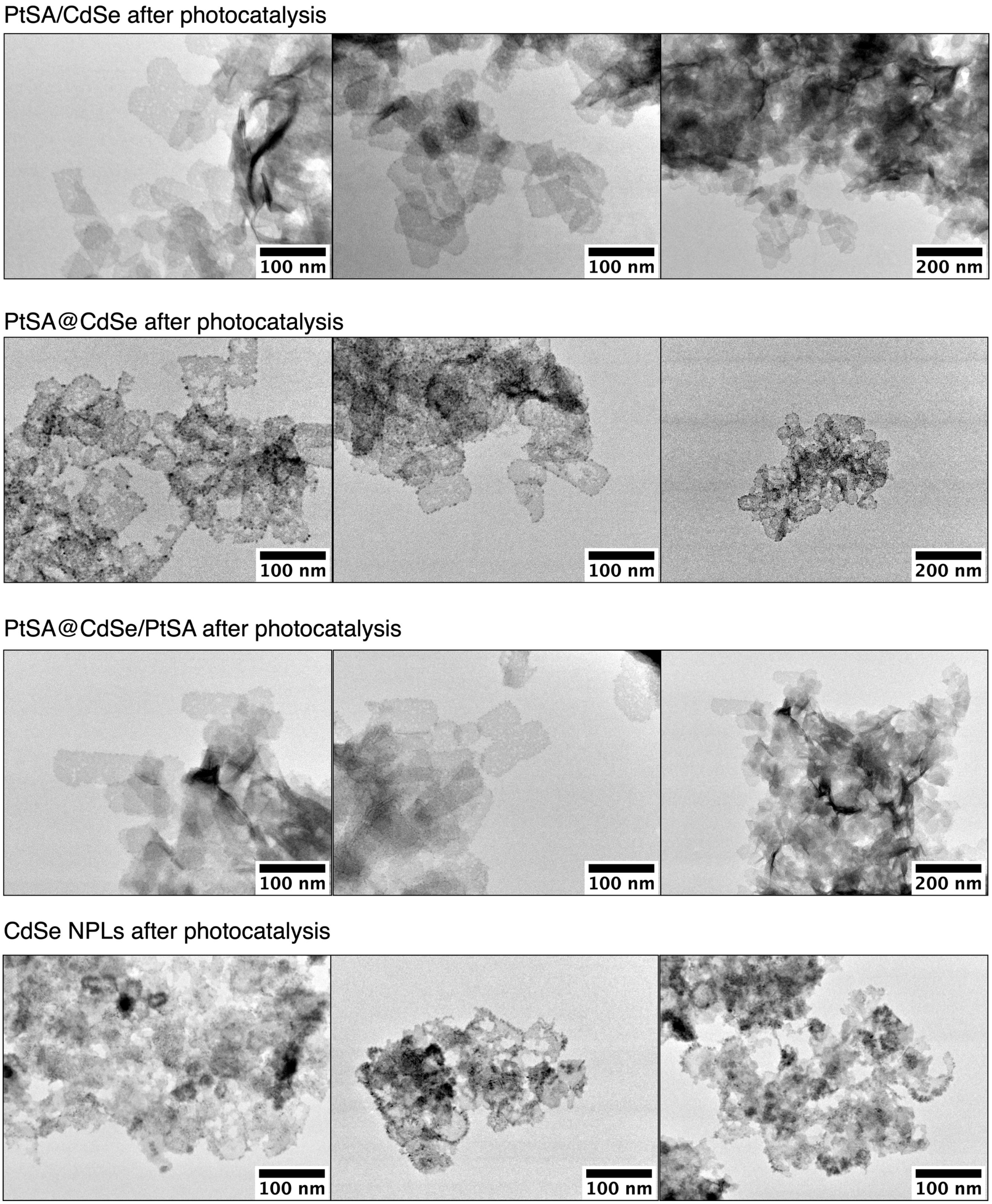


**Figure S35.** TEM images of catalysts recovered after photocatalysis.

**Table S7.** ICP–OES and SEM–EDX results of catalysts before and after photocatalysis. Cd/Se and Pt/Se atomic ratios were determined by ICP–OES. Cl/Se, S/Se, and K/Se atomic ratios were determined by a combination of ICP–OES (Cd/Se) and SEM–EDX (Cl/Cd, S/Cd, K/Cd). N.D.: not detected.

| Entry | Cd/Se | Pt/Se | Cl/Se | S/Se | K/Se |
|---|---|---|---|---|---|
| **PtSA/CdSe + MUA** | 1.010(10) | 0.175(2) | 0.0143(8) | 0.492(14) | 0.165(3) |
| **after photocatalysis** | 0.994(10) | 0.168(2) | 0.009(2) | 0.325(3) | N.D. |
| **PtSA@CdSe + $CdBr_2$ + MUA** | 1.203(12) | 0.0895(9) | | | |
| **after photocatalysis** | 1.087(5) | 0.0910(9) | 0.0181(11) | 0.360(5) | 0.0049(13) |
| **PtSA@CdSe/PtSA + MUA** | 0.945(9) | 0.250(2) | | | |
| **after photocatalysis** | 0.772(8) | 0.245(2) | 0.0067(9) | 0.329(13) | 0.0023(13) |
| **CdSe NPLs + $CdBr_2$ + MUA** | 1.208(12) | N.D. | N.D. | 0.312(7) | 0.048(4) |
| **after photocatalysis** | 1.108(10) | N.D. | 0.020(6) | 0.16(2) | 0.008(4) |

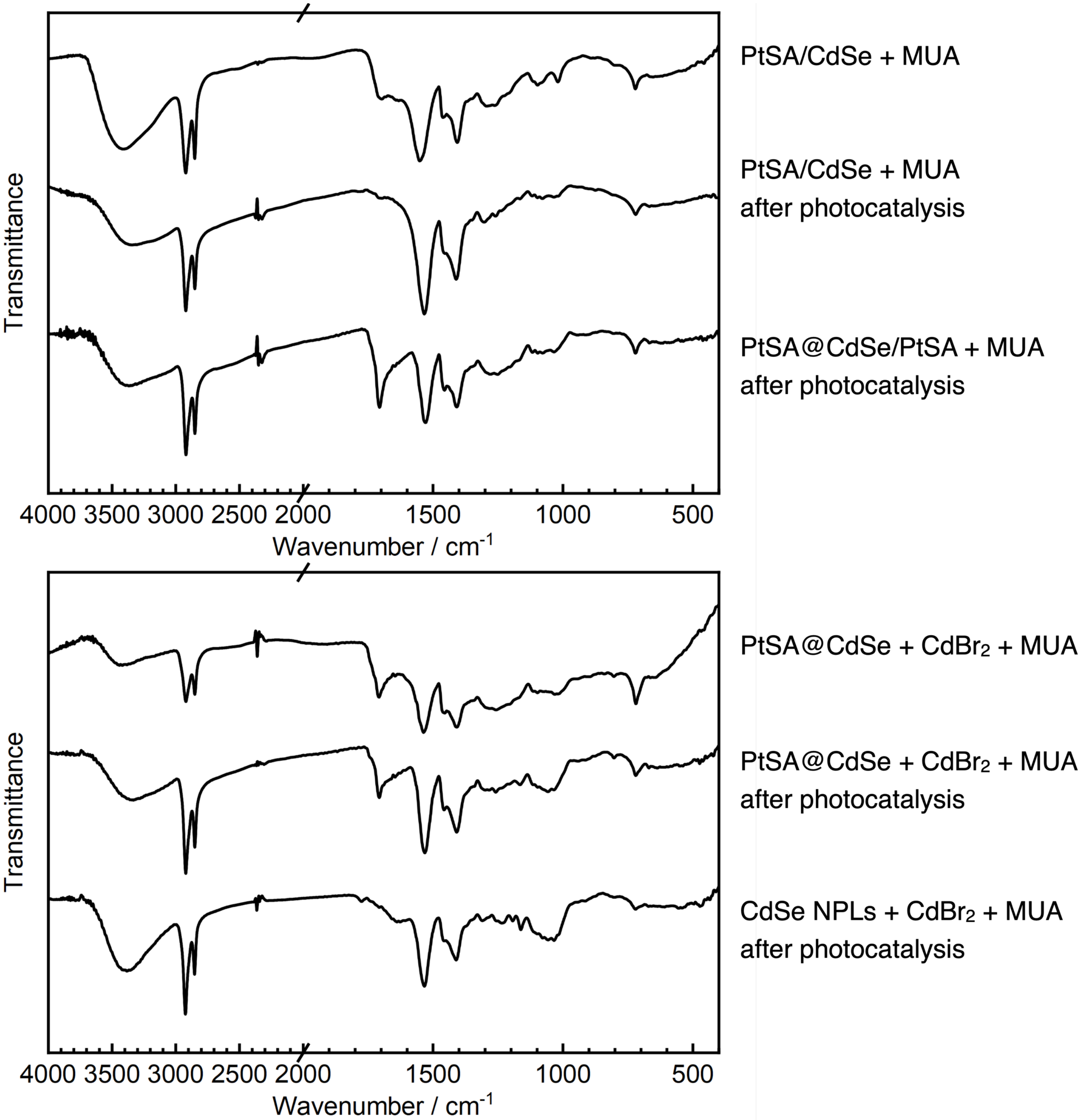


**Figure S36.** IR spectra of catalysts before and after photocatalysis. The appearance of the band around 1700 cm$^{-1}$ is attributable to the protonation of the carboxylate group in MUA during the catalyst recovery process.

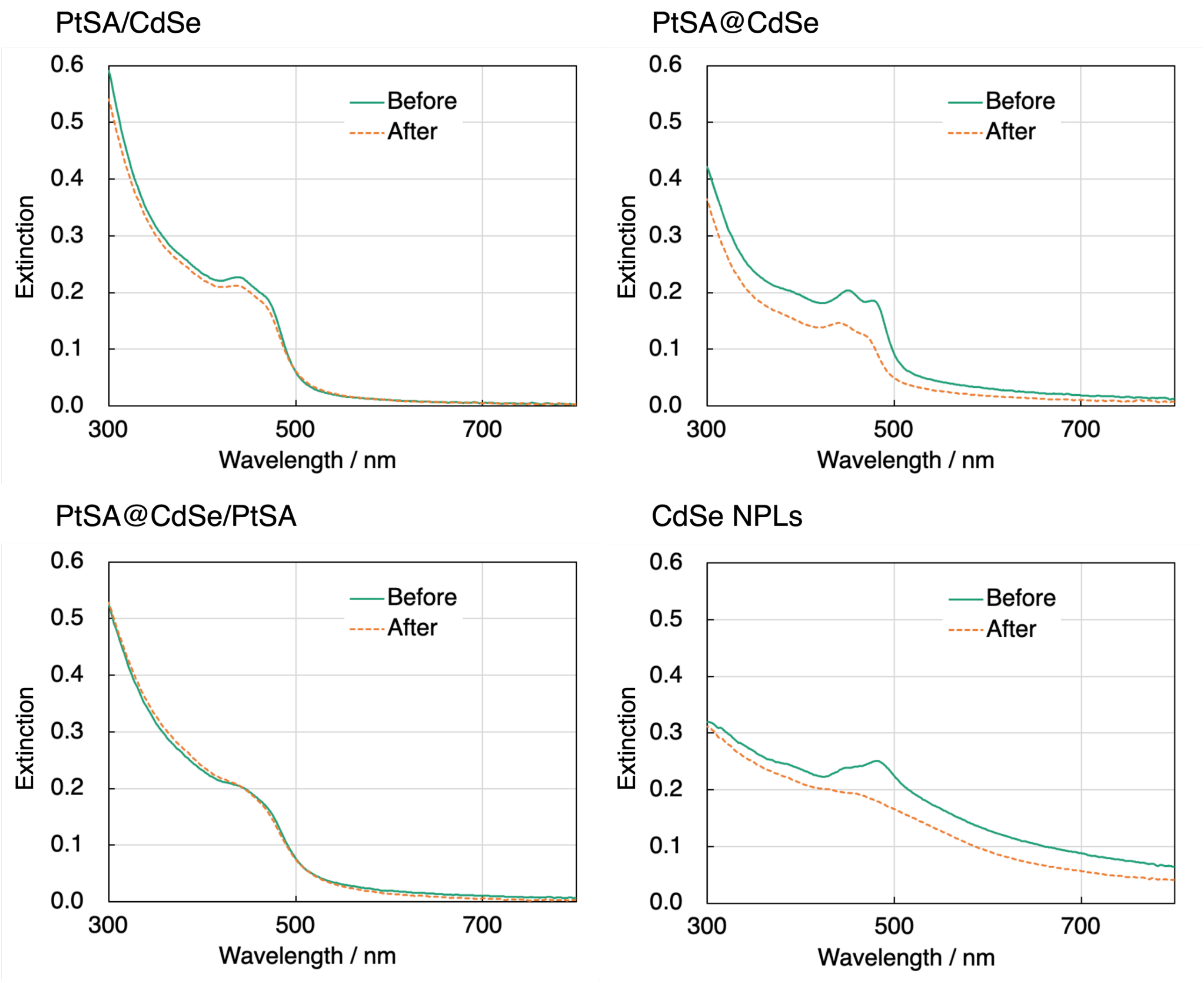


**Figure S37.** UV–Vis extinction spectra of catalyst dispersions before and after photoirradiation in $H_2O$/TEOA.

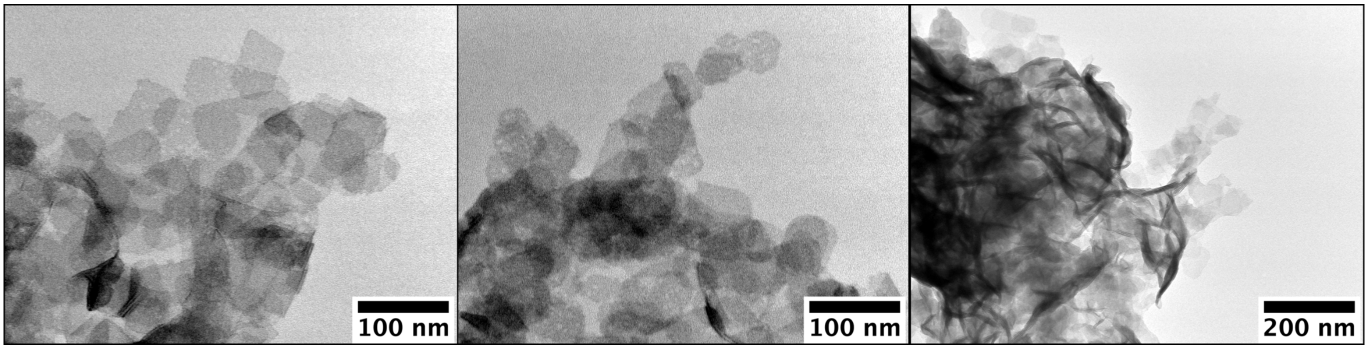


**Figure S38.** TEM images of PtSA/CdSe treated under the same conditions as those used for photocatalysis except for photoirradiation.

**Table S8.** Comparison of the photocatalytic hydrogen evolution activities of CdSe/Pt-based systems.

| Photocatalyst | Activity (mmol $h^{-1}$ $g^{-1}$) | Stability | Reference |
|---|---|---|---|
| **Single-semiconductor systems** | | | |
| PtSA/CdSe | 3.5 | Stable for 20 h | This work |
| PtSA@CdSe/PtSA | 2.4 | Stable for 5 h | This work |
| PtSA@CdSe | 1.8 | Unstable within 1 h | This work |
| PtNP/CdSe | 4.5 | Stable for 80 h | This work |
| Pt/CdSe | 5.1 | Not mentioned | [3] |
| Pt/CdSe | 3.4 | Not mentioned | [4] |
| Pt/CdSe | 1.9 | Unstable within 12 h | [5] |
| Pt/CdSe | 0.20 | Stable for 5 h | [6] |
| Pt/CdSe | 0.12 | Stable for 2 h | [7,8] |
| Pt/CdSe | 0.062 | Not mentioned | [9] |
| Pt/CdSe | 0.036 | Stable for 10 h | [10] |
| Pt/CdSe | 0 | Not applicable | [11] |
| **Heterojunction systems (not comprehensive)** | | | |
| (Cd,Ni)Se@(Cd,Ni)S | 150 | Stable for 10 h | [12] |
| CdSe@CdS/Pt | 40 | Stable for 12 h | [13] |
| Pt/CdSe/CN | 20 | Stable for 16 h | [3] |
| Pt/CdSe·DETA/ZnO | 11 | Stable for 18 h | [14] |
| CdSe@CdS/Pt | 0.43 | Not mentioned | [9] |

DETA = diethylenetriamine

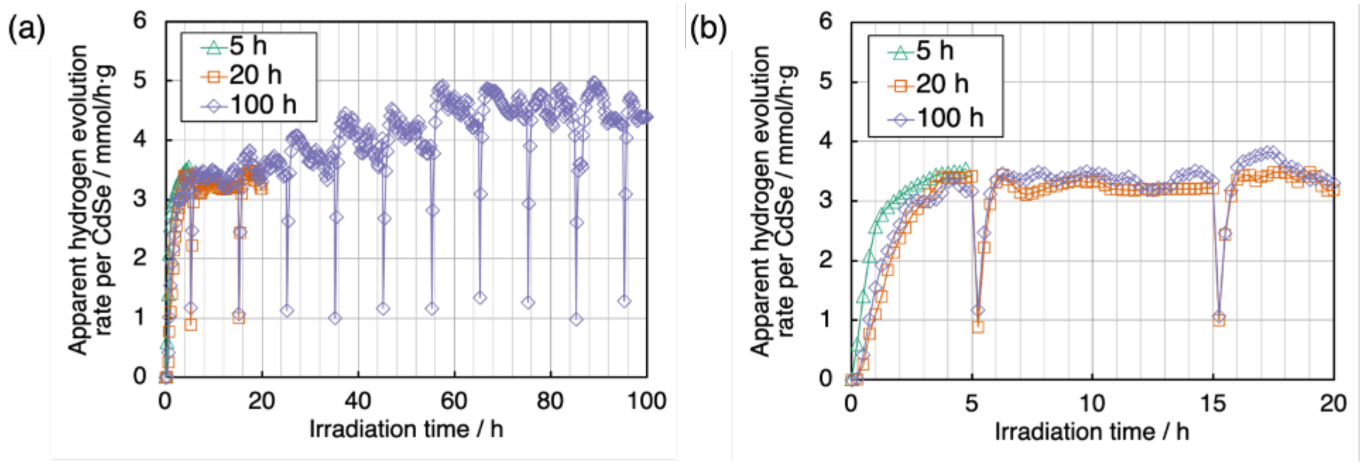


**Figure S39.** Hydrogen evolution rates with PtSA/CdSe for (a) 100 h and (b) initial 20 h. For the 20 h and 100 h runs, photoirradiation was intermittently interrupted after 5 h and every 10 h thereafter because of operation restrictions at night and on weekends. During the initial 1 h of each irradiation, the apparent hydrogen evolution rate measured by online GC shows a drop because the reactor headspace gas concentration has not yet reached a steady state.

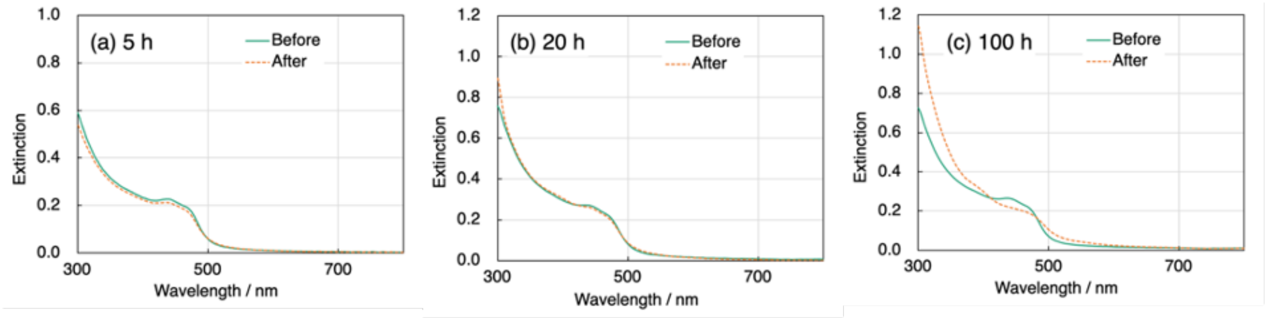


**Figure S40.** UV–vis extinction spectra of reaction mixtures before and after photocatalysis experiments with PtSA/CdSe: (a) 5 h, (b) 20 h, (c) 100 h runs.

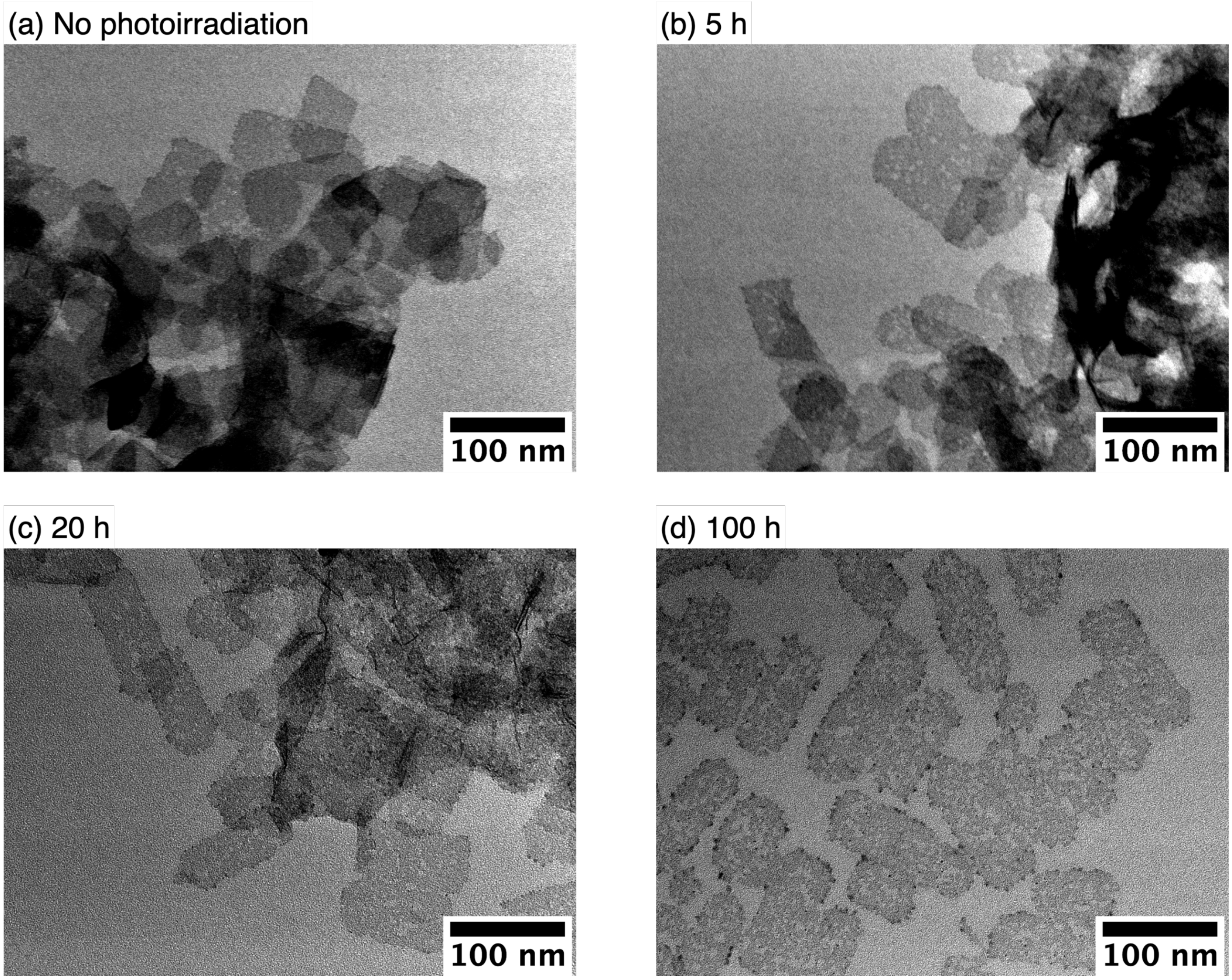


**Figure S41.** TEM images of catalysts recovered after photocatalysis experiments with PtSA/CdSe: (a) without photoirradiation for 5 h, (b–d) with photoirradiation for (b) 5 h, (c) 20 h, (d) 100 h.

# XAFS Analyses

## Determination of the amplitude reduction factor for FT-EXAFS curve-fitting

To conduct FT-EXAFS curve-fitting, we first analysed the data of a known compound *cis*-[$PtCl_2(dmso)_2$] to determine the amplitude reduction factor $S_0^2$ in our setup.

**Table S9.** FT-EXAFS curve-fitting parameters at the Pt L3 edge of *cis*-[$PtCl_2(dmso)_2$] at 10 K.

| Path | $N$ | $R$ (Å) | $\sigma^2$ ($10^{-3}$ Å$^2$) | $\Delta E_0$ (eV) | $S_0^2$ | R-factor ($10^{-3}$) |
|---|---|---|---|---|---|---|
| **S** | 2* | 2.223(12) | 0.7(18) | 7.5(11) | 0.86(8)§ | 6.76 |
| **Cl** | 2* | 2.315(11) | 0.3(16) | | | |

* Fixed according to the known structure to determine $S_0^2$.
§ Allowed to change during fitting.

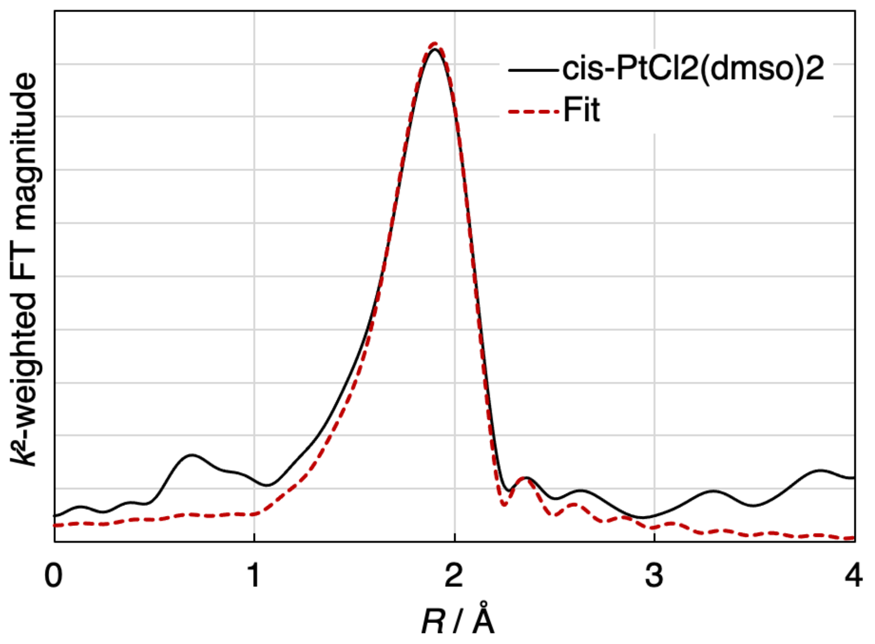


**Figure S42.** FT-EXAFS spectrum and fitted curve at the Pt L3 edge of *cis*-[$PtCl_2(dmso)_2$] at 10 K.

## FT-EXAFS of PtSA/CdSe

XAFS analysis of PtSA/CdSe shows decomposition during sample processing. The spectra vary with sample pellets. Especially, a longer grinding time causes an increase in Pt–Se peak intensity and a decrease in Pt–S/Cl peak intensity in FT-EXAFS spectra (Figure S43a). This result indicates that adsorbed Pt SAs substitutes Cd in CdSe under mechanical activation (Figure S43b). Since it is necessary to grind a sample to ensure the homogeneity required for XAFS, any obtained data contains a contribution of such decomposition.

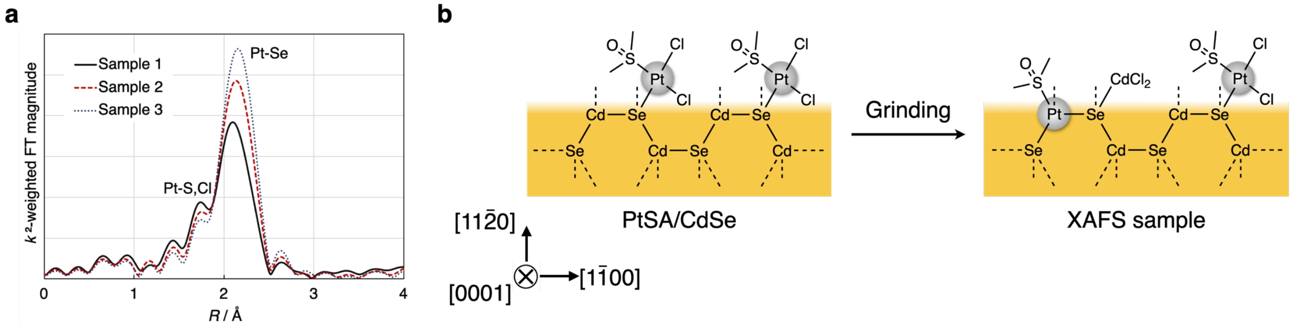


**Figure S43.** Decomposition of PtSA/CdSe during XAFS sample preparation. (a) FT-EXAFS spectra at the Pt L3 edge of PtSA/CdSe obtained by three different sample preparations. Sample 1: low humidity, grinding 40 min. Sample 2: high humidity, grinding 20 min. Sample 3: high humidity, grinding 80 min. (b) Schematic illustration of decomposition of PtSA/CdSe during grinding.

Keeping this decomposition in our mind, we conducted curve-fitting of the FT-EXAFS data of sample 1, the least decomposed sample (Table S8). Although the severe overlapping of S, Cl, and Se peaks makes it necessary to impose several constraints, a good fit is achieved when the partial substitution of Cd by Pt as in Table S8 is assumed (Figure S44). Namely, the Pt coordination environment in this sample can be explained by a combination of adsorbed Pt ($SCl_2Se$ coordination, 35%) and surface-substituting Pt ($SSe_3$ coordination, 65%). The true Pt coordination environment in PtSA/CdSe before the damage from grinding should be closer to the adsorbed state ($SCl_2Se$). Therefore, the FT-EXAFS fitting analysis partially supports our model structure with adsorbed Pt SAs, but it cannot rule out the possibility of partial substitution.

**Table S10.** FT-EXAFS curve-fitting parameters at the Pt L3 edge of PtSA/CdSe at 10 K.

| Path | $N$ | $R$ (Å) | $\sigma^2$ ($10^{-3}$ Å$^2$) | $\Delta E_0$ (eV) | $S_0^2$ | R-factor ($10^{-3}$) |
|---|---|---|---|---|---|---|
| **S** | 1[#] | 2.199(16) | 1.0(9)[$] | 5(2) | 0.86(8)[§] | 5.93 |
| **Cl** | 0.7(2)[#] | 2.33(2) | 0.4(4)[$] | | | |
| **Se** | 2.3(2)[#] | 2.439(6) | 2.3(3) | | | |

[§] Determined from the measurement of *cis*-[$PtCl_2(dmso)_2$].

[#] Constrained to follow the model structure in Figure S43b to avoid high correlations coming from severe overlapping. Namely, the coordination number of S is fixed to 1, while the other coordination numbers are constrained to be 3 in total.

[$] Constrained to be in the same ratio as in *cis*-[$PtCl_2(dmso)_2$] to avoid a high correlation coming from severe overlapping.

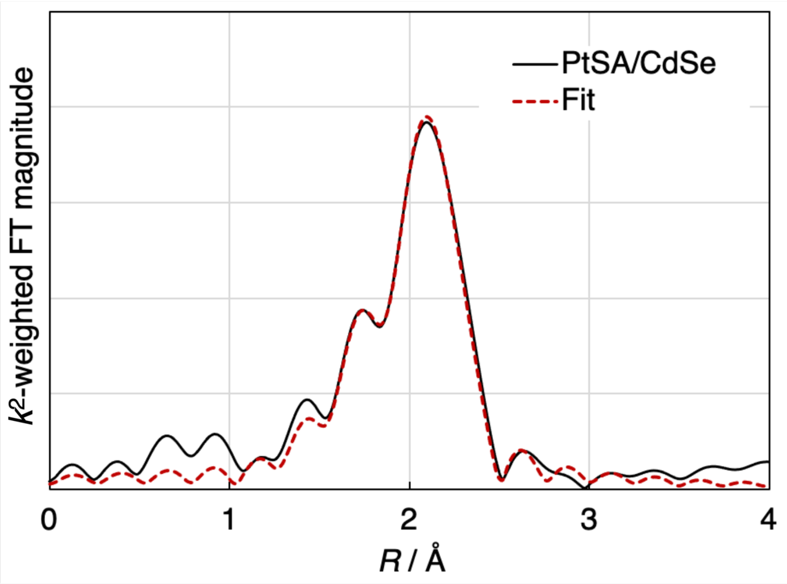


**Figure S44.** FT-EXAFS spectrum and the fitted curve at the Pt L3 edge of PtSA/CdSe at 10 K.

## FT-EXAFS of PtSA@CdSe

PtSA@CdSe is susceptible to similar sample damage because it contains some adsorbed Pt along with Pt substituting Cd in CdSe. The sample batch used for EXAFS had 51% of adsorbed Pt and 49% of substituted Pt, as estimated from ICP-OES and SEM-EDX analyses. The adsorbed Pt can turn into surface-substituting Pt during grinding (Figure S45).

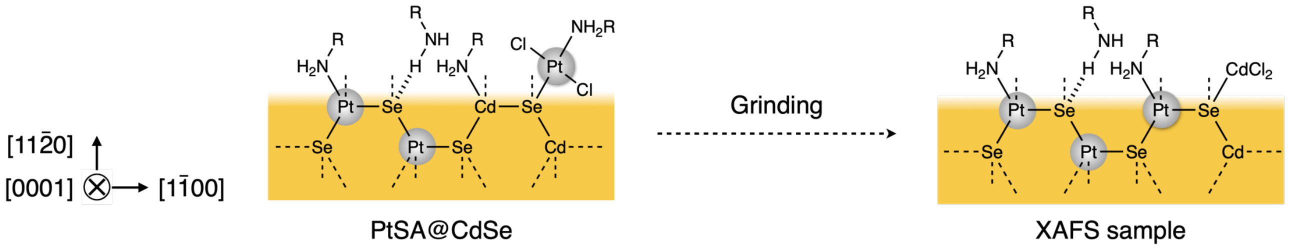


**Figure S45.** Schematic illustration of decomposition of PtSA@CdSe during grinding.

We attempted the FT-EXAFS curve-fitting at the Pt L3 edge of PtSA@CdSe. The FT-EXAFS spectrum of PtSA@CdSe shows a good fit with the parameters shown below (Table S11, Figure S46). The coordination environment of Pt in this sample can be explained by a combination of adsorbed ($NCl_2Se$, 0–50% as a 95% confidence interval), surface-substituting ($NSe_3$, 30–100%), and interior-substituting ($Se_4$, 0–70%). Because of the large uncertainties and the possible sample damages, this result cannot fully validate our model structure.

**Table S11.** FT-EXAFS curve-fitting parameters at the Pt L3 edge of PtSA@CdSe at RT.

| Path | *N* | *R* (Å) | $\sigma^2$ ($10^{-3}$ Å$^2$) | $\Delta E_0$ (eV) | $S_0^2$ | R-factor ($10^{-3}$) |
|---|---|---|---|---|---|---|
| **N** | 0.9(3)* | 2.11(4) | 2(4) | | | |
| **Cl** | 0.2(4)* | 2.309$ | 0.63(12) | 9(3) | 0.86(8)§ | 10.4 |
| **Se** | 2.9(3)* | 2.449(10) | 3.6(6) | | | |

§ Determined from the measurement of *cis*-[$PtCl_2$(dmso)$_2$].
* Constrained to be 4 in total to avoid high correlations coming from severe overlapping.
$ Fixed to be the same as the average Pt–Cl distance in the reported single-crystal structure of *cis*-[$PtCl_2$(dmso)$_2$] to avoid having too many fitting parameters.[15]

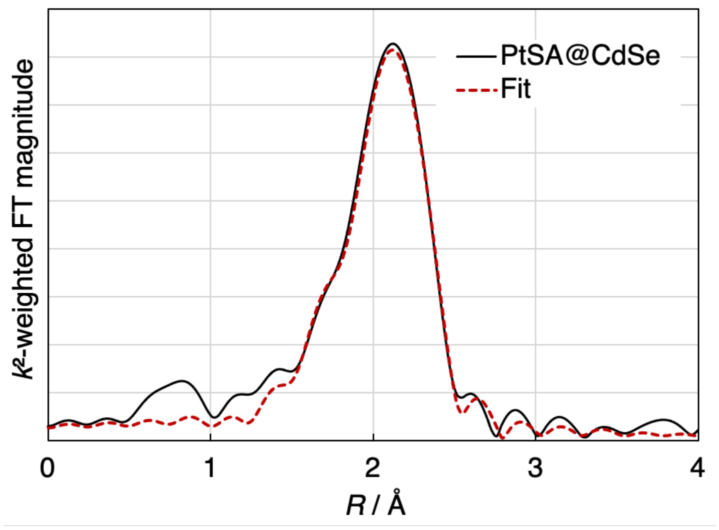


**Figure S46.** FT-EXAFS spectrum and fitted curve of PtSA@CdSe at the Pt L3 edge at RT.

## XANES

XANES analysis also gives clues to the coordination environment of Pt. It is known that a Pt L3 edge shows a "white line" peak whose intensity and shape depend on its valence and coordination species.[16,17] Typically, more positively charged Pt exhibits a stronger white line. Here, the XANES spectra of PtSA/CdSe and PtSA@CdSe show white lines with intensities higher than that of Pt foil and similar to *cis*-[$PtCl_2(dmso)_2$], indicating that the Pt atoms in PtSA/CdSe and PtSA@CdSe have $Pt^{II}$ states (Figure S47). The differences in the shapes of white lines can be assigned to differences in the coordination environments, but the coordination environments are affected by the sample damage discussed in the FT-EXAFS fitting above. The uncontrollable sample damage as well as the complicated effects from each coordination species (dmso, Cl, Se, amines) preclude further analysis of XANES spectra.

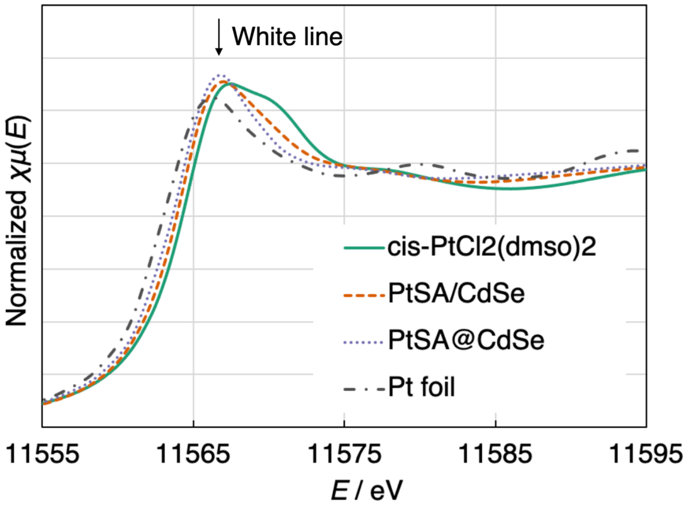


**Figure S47.** XANES spectra at the Pt L3 edge (for the normalization method, see the Methods section of the main text for the normalization method).

In conclusion, FT-EXAFS curve-fitting and XANES analyses are not reliable enough to confirm the validity of the proposed models of PtSA/CdSe and PtSA@CdSe because of the sample damages during sample processing. Nonetheless, the combination of other characterization techniques comprehensively supports our model structures.

## Supplementary References


1. Risberg, E. D. et al. *Dalton Trans.*, 1328-1338 (2009).
2. Cotton, F. A., Francis, R. & Horrocks, W. D. *J. Phys. Chem.* **64**, 1534-1536 (1960).
3. Wang, Y., Wang, H., Li, Y., Zhang, M. & Zheng, Y. *Molecules* **27** (2022).
4. Kim, W. D. et al. *Chem. Mater.* **28**, 962-968 (2016).
5. Hu, T. et al. *J. Colloid Interface Sci.* **555**, 166-173 (2019).
6. Frame, F. A. & Osterloh, F. E. *J. Phys. Chem. C* **114**, 10628-10633 (2010).
7. Sung, Y. et al. *CrystEngComm* **17**, 8423-8427 (2015).
8. Karakus, M. et al. *J. Phys. Chem. C* **121**, 13070-13077 (2017).
9. Tongying, P. et al. *Nanoscale* **6**, 4117-4124 (2014).
10. Bang, J. U., Lee, S. J., Jang, J. S., Choi, W. & Song, H. *J. Phys. Chem. Lett.* **3**, 3781-3785 (2012).
11. Li, Q. et al. *J. Am. Chem. Soc.* **140**, 11726-11734 (2018).
12. Li, Z. J. et al. *Adv. Mater.* **25**, 6613-6618 (2013).
13. Amirav, L. & Alivisatos, A. P. *J. Phys. Chem. Lett.* **1**, 1051-1054 (2010).
14. Li, Z., Jin, D. & Wang, Z. *Appl. Surf. Sci.* **529** (2020).
15. Melanson, R. & Rochon, F. D. *Can. J. Chem.* **53**, 2371-2374 (1975).
16. Mansour, A. N., Cook, J. W. & Sayers, D. E. *J. Phys. Chem.* **88**, 2330-2334 (2002).
17. Ankudinov, A. L., Rehr, J. J., Low, J. J. & Bare, S. R. *Top. Catal.* **18**, 3-7 (2002).
